# Structure-function analysis of the AMPK activator SC4 and identification of a potent pan AMPK activator

**DOI:** 10.1042/BCJ20220067

**Published:** 2022-06-08

**Authors:** Ashley J. Ovens, Yi Sing Gee, Naomi X.Y. Ling, Dingyi Yu, Justin P. Hardee, Jin D. Chung, Kevin R.W. Ngoei, Nicholas J. Waters, Nolan J. Hoffman, John W. Scott, Kim Loh, Katrin Spengler, Regine Heller, Michael W. Parker, Gordon S. Lynch, Fei Huang, Sandra Galic, Bruce E. Kemp, Jonathan B. Baell, Jonathan S. Oakhill, Christopher G. Langendorf

**Affiliations:** 1 Metabolic Signalling Laboratory, St. Vincent's Institute of Medical Research, Fitzroy 3065, Australia; 2 Exercise and Nutrition Research Program, Mary MacKillop Institute for Health Research, Australian Catholic University, Melbourne 3000, Australia; 3 Medicinal Chemistry, Monash Institute of Pharmaceutical Sciences, Monash University, Parkville 3052, Australia; 4 Protein Chemistry and Metabolism, St. Vincent's Institute of Medical Research, Fitzroy 3065, Australia; 5 Centre for Muscle Research, Department of Anatomy and Physiology, The University of Melbourne, Melbourne, Victoria 3010, Australia; 6 The Florey Institute of Neuroscience and Mental Health, Royal Parade, Parkville 3052, Australia; 7 Institute of Molecular Cell Biology, Center for Molecular Biomedicine, Jena University Hospital, 07745 Jena, Germany; 8 ACRF Rational Drug Discovery Centre, St. Vincent's Institute of Medical Research, Fitzroy 3065, Australia; 9 Structural Biology and Computational Design Laboratory, Department of Biochemistry and Pharmacology, Bio21 Molecular Science and Biotechnology Institute, University of Melbourne, Parkville, Victoria, Australia; 10 School of Pharmaceutical Sciences, Nanjing Tech University, No. 30 South Puzhu Road, Nanjing 211816, People's Republic of China

**Keywords:** AMPK, drug discovery and design, metabolic disorders, structural biology, type 2 diabetes

## Abstract

The AMP-activated protein kinase (AMPK) αβγ heterotrimer is a primary cellular energy sensor and central regulator of energy homeostasis. Activating skeletal muscle AMPK with small molecule drugs improves glucose uptake and provides an opportunity for new strategies to treat type 2 diabetes and insulin resistance, with recent genetic and pharmacological studies indicating the α2β2γ1 isoform combination as the heterotrimer complex primarily responsible. With the goal of developing α2β2-specific activators, here we perform structure/function analysis of the 2-hydroxybiphenyl group of SC4, an activator with tendency for α2-selectivity that is also capable of potently activating β2 complexes. Substitution of the LHS 2-hydroxyphenyl group with polar-substituted cyclohexene-based probes resulted in two AMPK agonists, MSG010 and MSG011, which did not display α2-selectivity when screened against a panel of AMPK complexes. By radiolabel kinase assay, MSG010 and MSG011 activated α2β2γ1 AMPK with one order of magnitude greater potency than the pan AMPK activator MK-8722. A crystal structure of MSG011 complexed to AMPK α2β1γ1 revealed a similar binding mode to SC4 and the potential importance of an interaction between the SC4 2-hydroxyl group and α2-Lys31 for directing α2-selectivity. MSG011 induced robust AMPK signalling in mouse primary hepatocytes and commonly used cell lines, and in most cases this occurred in the absence of changes in phosphorylation of the kinase activation loop residue α-Thr172, a classical marker of AMP-induced AMPK activity. These findings will guide future design of α2β2-selective AMPK activators, that we hypothesise may avoid off-target complications associated with indiscriminate activation of AMPK throughout the body.

## Introduction

AMP-activated protein kinase (AMPK) is a conserved serine/threonine protein kinase that acts as a metabolic fuel sensor and is crucial for maintaining cellular energy homeostasis [1,2]. It is a heterotrimer composed of a catalytic α subunit, an N-terminally myristoylated regulatory β subunit and a nucleotide-sensing γ subunit, each with multiple isoforms (α1, α2, β1, β2, γ1, γ2, γ3) providing the potential to form 12 distinct complexes. The capacity of AMPK to monitor cellular energy status is provided by three exchangeable adenine nucleotide binding sites on the γ-subunit. Increases in AMP/ATP and ADP/ATP adenylate ratios arising from energy stress cause either allosteric activation or increases in phosphorylation on the α-subunit activation loop residue threonine-172 (α-Thr172) by liver kinase B1 (LKB1) or calcium/calmodulin-dependent kinase kinase 2 (CaMKK2) [3,4]. Under conditions of energy or nutrient stress, AMPK directs metabolism towards ATP-conserving (catabolic) pathways and away from ATP-consuming (anabolic) pathways. It does this by directly phosphorylating and regulating rate-limiting enzymes, and transcription factors regulating their expression, in multiple key biochemical pathways such as fatty acid oxidation and synthesis, mitochondrial biogenesis, skeletal muscle glucose uptake, cholesterol synthesis and autophagy. These pleiotropic effects place AMPK as a promising drug target for the treatment of diseases such as type 2 diabetes mellitus (T2DM), metabolic syndrome, cancer, neurodegeneration and cardiovascular disease [5–8]. Indeed, some of the pleiotropic effects of the biguanide metformin, the first line treatment for T2DM, have been attributed to AMPK-dependent mechanisms through inhibition of complex 1 in the mitochondrial electron transport chain [9,10]. This leads to impaired ATP production and a consequent increase in AMP/ATP ratio, resulting in canonical nucleotide-dependent activation of AMPK primarily through increased α-Thr172 phosphorylation. Indirect AMPK activation is a hallmark of a large group of natural and synthetic agents that trigger AMPK signalling by inhibiting either mitochondrial function or glycolysis to induce metabolic stress [5].

Recent progress has been made in developing small-molecule, allosteric activators that directly bind to AMPK. Biochemical and structural analyses reveal that many of these direct activators bind to a hydrophobic pocket located between the AMPK α kinase domain and β carbohydrate binding module (CBM), termed the Allosteric Drug and Metabolite (ADaM) site, as it can also be occupied by regulatory endogenous metabolites such as palmitoyl-CoA [11,12]. Direct synthetic activators can be sub-divided into those specific for AMPK complexes containing the β1 isoform (β1AMPK) (e.g. A-769662, salicylate, MT47-100) [13–16], ‘pan’ activators capable of activating all 12 possible AMPK heterotrimer combinations (e.g. MK-8722 and most likely PF-739 and I-3-40) [17–19] and intermediate activators that display a degree of isoform preference (e.g. 991 (β1 > β2), SC4 (α2 > α1) and C2/C13 (γ1/2 > γ3) [20–22]. The 991 derivative R739 is an anomaly in that it does not allosterically activate α1β2γ1 AMPK in cell free assays but promotes β2AMPK signalling in HepG2 cells, reportedly as a consequence of being able to protect α-pThr172 from dephosphorylation by protein phosphatases [23], a feature described biochemically for AMP and most compounds targeting the ADaM site.

AMPK isoforms display tissue-specific expression profiles therefore activator isoform selectivity raises opportunities to preferentially target subsets of AMPK complexes. α1, β1 and γ1 isoforms are found in multiple human tissues while α2β2γ1 and α1β2γ1 together make up the majority of AMPK complexes in skeletal muscle [24]. Expression of γ2 is predominantly limited to the heart, whereas γ3 is found almost exclusively complexed to ∼30% of α2β2 complexes in glycolytic skeletal muscle. The importance of activator selectivity to prevent indiscriminate activation throughout the body is demonstrated by the pan AMPK activator MK-8722, which induced robust improvements in key disease hallmarks, such as systemic glucose clearance and improved glycaemic control, through activation of β2AMPK in skeletal muscle of rodent and non-human primate models of T2DM [17]. However, MK-8722 also induced reversible cardiac hypertrophy in both lean and diabetic animal models, attributed to increased ventricular wall area and glycogen content. Although the investigation into off-tissue target effects of MK-8722 was not reported, it can be inferred that MK-8722 induced hypertrophy resulted from activation of AMPK in the heart, since activating mutations in the AMPK γ2 subunit, expressed in the heart, are linked to excessive cardiac glycogen accumulation in Wolff–Parkinson–White syndrome [25–28].

Recent *in vivo* studies have shown that MK-8722, PF-739 and 991 induced skeletal muscle glucose uptake independently of α2β2γ3 [29,30]. This important finding, combined with our previous report showing β2-dependent increases in *ex vivo* glucose uptake in mouse soleus and extensor digitorum longus (EDL) muscles by the imidazopyridine SC4, implicate α2β2γ1 complexes as the physiological target for ADaM site drugs in skeletal muscle [21,29,30]. Furthermore, SC4 activates all six α2 complexes *in vitro* but demonstrates poor activation potential against α1β2γ1. Our study, supported by computational analysis of the molecular interaction networks involved, demonstrated the importance of the β2-specific residue Asp111 and the SC4 imidazopyridine 4-nitrogen atom for β2 activation by SC4, however we know very little regarding the determinants mediating apparent α2 selectivity [21,31]. Here we describe preliminary SAR analysis of the SC4 phenylphenol and reveal its contribution to the α isoform discriminating properties of this compound. Our findings will aid efforts to develop clinically viable, glucose-controlling drugs through specific activation of α2β2γ1 in skeletal muscle.

## Results

### SC4 derivatives demonstrate pan AMPK isoform activation

**Figure 1.**
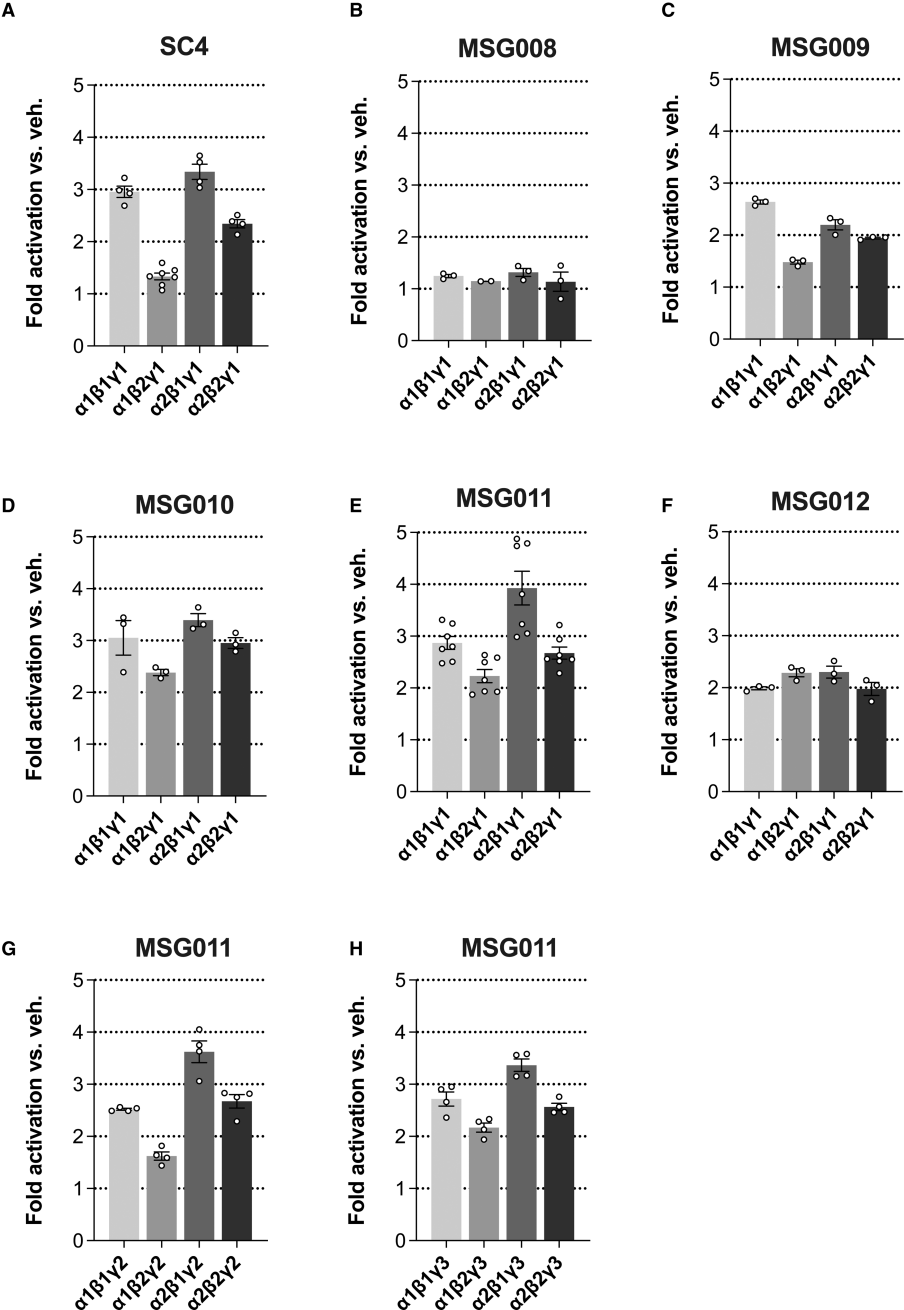
Comparative *in vitro* allosteric activation of AMPK complexes. GST-tagged AMPK from HEK293T/17 cells was immobilised on glutathione Sepharose and activities measured by SAMS assay ± 1 µM compounds. Allosteric activation of human γ1 AMPK complexes by (**A**) SC4, (**B**) MSG008, (**C**) MSG009, (**D**) MSG010, (**E**) MSG011 and (**F**) MSG012. Allosteric activation of human (**G**) γ2 and (**H**) γ3 AMPK complexes by MSG011. *n* = 2–7, data presented as mean fold AMPK activation relative to vehicle ± SEM.

We generated a series of compounds to investigate whether modification of the SC4 2-hydroxybiphenyl LHS fragment affects isoform selectivity, some of which (MSG008-MSG011) are presented in Table 1. We also synthesised compound MSG012 (example I-3-54 [32]), which was reported to activate α2β2γ1 with ∼100-fold greater potency compared with α1β1γ1. MSG012 differs from SC4 by substitution of the 2-hydroxyphenyl LHS fragment with 3-cyclohexen-4-yl-1-carboxylic acid, and 2-methylbenzoic acid RHS fragment with 1-hydroxy-N,N-dimethylcyclohexan-4-yl-1-carboxamide. Our initial screen involved measuring the activity of recombinant human GST-AMPK complexes expressed in HEK293T/17 cells and immobilised on glutathione-Sepharose (Supplementary Figure S1) [33]. Enzyme activity was measured by radiolabelling the synthetic SAMS peptide substrate in the absence or presence of 1 µM compound. Consistent with our previous characterisation, SC4 activated α1β1γ1, α2β1γ1 and α2β2γ1 >2.3-fold, and weakly activated α1β2γ1 1.3-fold (Figure 1A) [21]. MSG008 failed to activate all γ1 AMPK complexes above 1.3-fold (Figure 1B). MSG009 activated α1β1γ1, α2β1γ1 and α2β2γ1, albeit with reduced fold activation relative to SC4, and activated α1β2γ1 1.5-fold (Figure 1C). Interestingly, both MSG010 (Figure 1D) and MSG011 (Figure 1E), which differ from SC4 by substitution of the 2-hydroxyphenyl group for 3-cyclohexen-4-yl-1-carboxylic acid or *N*-(3-cyclohex-3-en-4-yl)acetamide, respectively, activated all γ1 complexes >2.2-fold, whereas MSG012 activated all γ1 complexes to a similar extent (between 2- and 2.3-fold) (Figure 1F). The γ isoform had little influence on AMPK activation by MSG011 as most γ2 and γ3 complexes were activated >2.2-fold at 1 µM, with α1β2γ2 being the only exception (1.6-fold activation). Combined, our data indicate the LHS terminal phenyl group of SC4 is a critical determinant for AMPK activation and its 2-hydroxy group may be important for determining α isoform selectivity. Accordingly, MSG010, MSG011 and MSG012 can be classified as pan AMPK activators.

**Table 1:** Structures of SC4 analogues used in this study

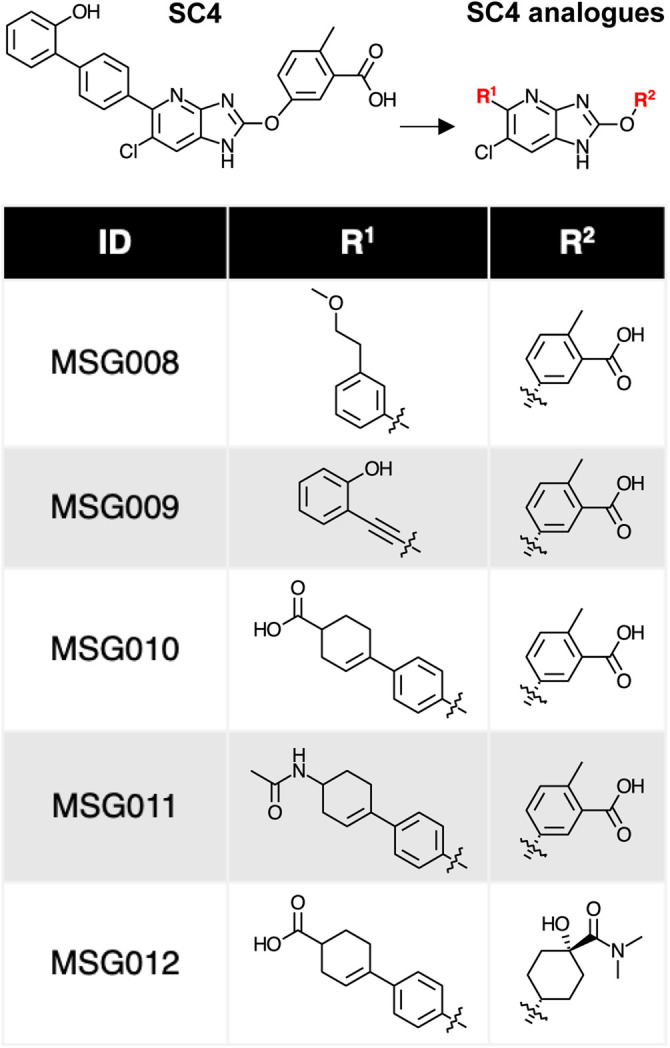

### Biochemical analysis of pan AMPK activators

**Figure 2.**
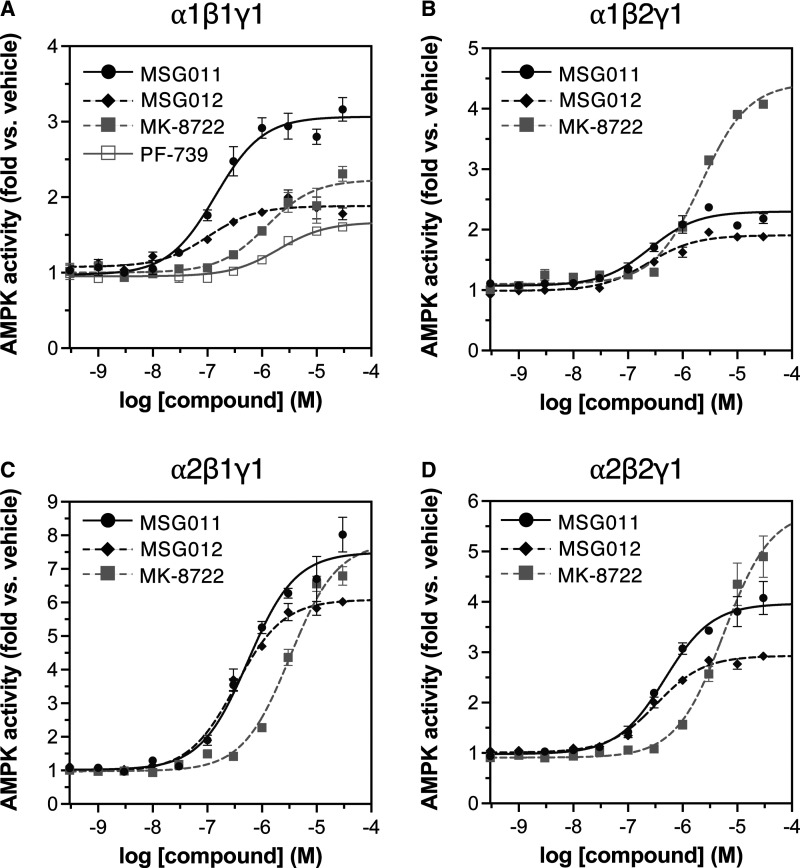
Activation kinetics for γ1 AMPK complexes. GST-tagged AMPK from HEK293T/17 cells was immobilised on glutathione Sepharose and activities measured by SAMS assay in the presence of 0–30 µM AMPK activators. Dose-response curves for MSG011, MSG012, MK-8722 and PF-739 activation of (**A**) α1β1γ1, (**B**) α1β2γ1, (**C**) α2β1γ1 and (**D**) α2β2γ1. *n* = 3–6, data presented as mean fold AMPK activation relative to vehicle ± SEM. See Supplementary Table S2.

We directly compared *in vitro* potencies of pan activators by performing radiolabel kinase assays with γ1AMPK complexes. Initially, we used GST-tagged γ1AMPK expressed in HEK293T/17 cells and immobilised on glutathione-Sepharose (Figure 2 and Table 2). Calculated EC_50_ values for MSG012 activation were 103.1 nM with α1β1γ1 and 328.0 nM with α2β2γ1, thus we were unable to demonstrate greater potency for β2 over β1AMPK that was previously described for this compound [32]. EC_50_ values for MSG011 ranged from 140.4 nM (α1β1γ1) to 553.4 nM (α2β1γ1). Under these assay conditions, EC_50_ values for γ1AMPK activation by MK-8722 (1.2–5.6 µM) and PF-739 (2.0 µM) were 100–1000-fold higher than previously determined by high-throughput, fluorescence-based assays performed in solution (Table 2) [17,18]. These assays used AMPK purified from Sf9 insect cells or *E. coli* that was pre-treated with CaMKK2. Reduced potency of MK-8722 in our assays was not due to quality of the compound, which we validated by NMR (Supplementary Figure S2) [34], nor was it due to steric hindrance caused by AMPK immobilisation since GST-α1β1γ1 in solution demonstrated similar activation kinetics (Supplementary Figure S3A). However, relative to immobilised GST-α2β1γ1 from HEK293T/17 cells, His-tagged and myristoylated α2β1γ1 AMPK (expressed in *E. coli*, CaMKK2-treated and assayed in solution) was at least 10-fold more sensitive to activation by both MK-8722 and MSG011 (Supplementary Figure S3B), indicating the GST-affinity tag or CaMKK2 treatment may exert some influence on sensitivity to these activators.

**Table 2:** Parameters for compound activation of immobilised γ1 AMPK complexes

	α1β1γ1		α1β2γ1		α2β1γ1		α2β2γ1	
Compound	EC_50_ (nM)	Max. fold activation	EC_50_ (nM)	Max. fold activation	EC_50_ (nM)	Max. fold activation	EC_50_ (nM)	Max. fold activation
MSG011	140.4	3.1	248.9	2.3	553.4	7.5	472.9	4.0
MSG012	103.1	1.9	232.1	1.9	331.8	6.1	328.0	2.9
MK-8722	1207.8	2.2	2182.7	4.4	3250.9	7.8	5597.6	5.8
PF-739	2009.1	1.7	n.d.	n.d.	n.d.	n.d.	n.d.	n.d.
SC4^1^	5.1	2.9	28.6^2^	1.4	n.d.	n.d.	17.2	2.5

1 Previously determined in solution using mammalian cell-expressed AMPK [21];

2 *R*^2^ for curve fit = 0.773 (GraphPad).

MK-8722 at concentrations below 1 µM was previously reported to stimulate AMPK signalling in HepG2 cells and C2C12 myotubes in the absence of increased α-Thr172 phosphorylation [17], which is often used as a marker of AMPK activation by compounds targeting the allosteric ADaM site. MK-8722 above 1 µM elevated α-Thr172 in these cells, although this did not translate to further robust increases in phosphorylation of ACC. To explore this dose-dependent response, we used HEK293T/17 cells transiently expressing WT γ1AMPK or the AMP-insensitive γ1 mutant R299G (Supplementary Figure S4A). Phosphorylation of α-Thr172 was significantly increased in WT γ1-expressing cells by 2.5 µM MK-8722, however the effect was largely abrogated in γ1-R299G-expressing cells. One explanation is that 2.5 µM MK-8722 caused indirect activation of AMPK (α-Thr172 phosphorylation) by inducing a metabolic stress leading to rises in AMP/ATP and ADP/ATP ratios. We examined the effect of MK-8722 incubation on C2C12 mitochondrial function (Supplementary Figure S4B–I). Incubation with 10 µM, but not 1 µM, MK-8722 significantly impaired basal respiration, ATP production, maximal respiration, spare respiratory capacity, non-mitochondrial oxygen consumption and coupling efficiency. Impaired respiration was not due to loss of cell viability was determined by total protein stain (Supplementary Figure S4J).

### MSG011 activation of AMPK in cells occurs primarily via an allosteric mechanism

**Figure 3.**
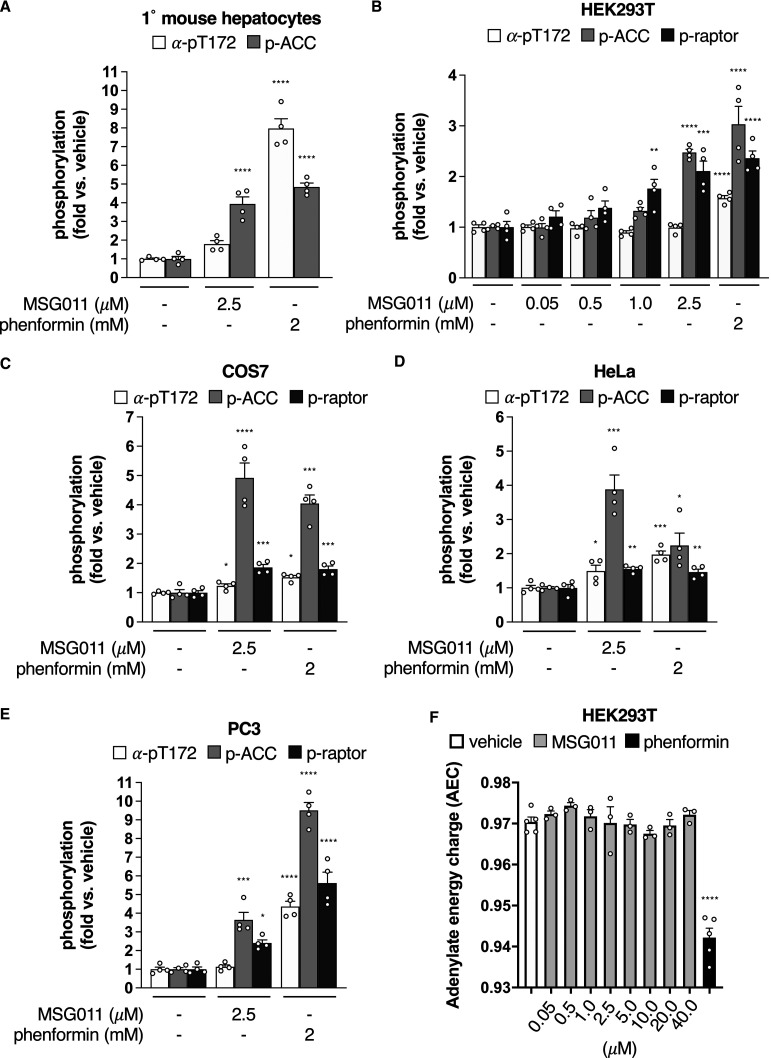
Bioactivity of MSG011 in cultured cells. (**A**) Mouse primary hepatocytes, (**B**) HEK293T, (**C**) COS7, (**D**) HeLa (cell model of cervical cancer) and (**E**) PC3 (cell model of prostate cancer) were treated with MSG011 or phenformin as indicated and cell lysates assessed for phosphorylation of AMPKα-Thr172, ACC-Ser79 and raptor-Ser792 by immunoblot. *n* = 4, data presented as mean fold change in phosphorylation relative to untreated ± SEM. Statistical analysis was performed by one-way ANOVA with post hoc Dunnett's multiple comparison test. * *P* < 0.05, ** *P* < 0.01, *** *P* < 0.001 and **** *P* < 0.0001 indicate significant increase in phosphorylation relative to vehicle. (**F**) HEK293T cells were treated with MSG011 (as indicated, 1 h) or phenformin (2 mM, 1 h) and cell lysates assessed for adenine nucleotides measured by LC–MS. *n* = 3–5, data presented as mean adenylate energy charge ± SEM. Statistical analysis was performed by one-way ANOVA with post hoc Dunnett's multiple comparison test. **** *P* < 0.0001 indicates significant reduction in adenylate energy charge relative to vehicle. Antibodies used are described in Supplementary Table S3.

To evaluate the efficacy of MSG011 *in cellulo*, we incubated mouse primary hepatocytes with 2.5 µM MSG011 for 60 min and tracked changes in phosphorylation of AMPK α-Thr172 (marker of activation) and the AMPK substrate acetyl-CoA carboxylase (ACC) Ser79 (AMPK signalling marker). Both MSG011 and phenformin, a mitochondrial complex I inhibitor that indirectly activates AMPK by dramatically depleting cellular adenylate energy charge (AEC), induced similar increases in phosphorylation of ACC-Ser79 (Figure 3A and Supplementary Figure S5A). However, unlike phenformin, MSG011 activation of AMPK signalling in hepatocytes occurred independently of significant net phosphorylation of AMPK α-Thr172. MSG011 (2.5 µM) also triggered AMPK signalling (measured by phosphorylation of ACC-Ser79 and the alternate AMPK substrate raptor-Ser792) at levels comparable to extreme energy stress in HEK293T and COS7 cell lines, and significantly activated AMPK in commonly used cancer cell lines HeLa and PC3 (Figure 3B–E and Supplementary Figure S5B–E). In HEK293T and PC3 cells these effects were independent of changes to α-Thr172 phosphorylation. Using LC–MS to directly measure relative cellular levels of AMP, ADP and ATP [16], we found that AEC was unaffected by incubation of HEK293T cells with MSG011 up to 40 µM (Figure 3F), indicating that activation of AMPK by this compound was not due to elevated cellular ADP or AMP concentrations.

### Structural comparison of AMPK/drug complexes reveals binding determinants potentially important for α2 selectivity

**Figure 4.**
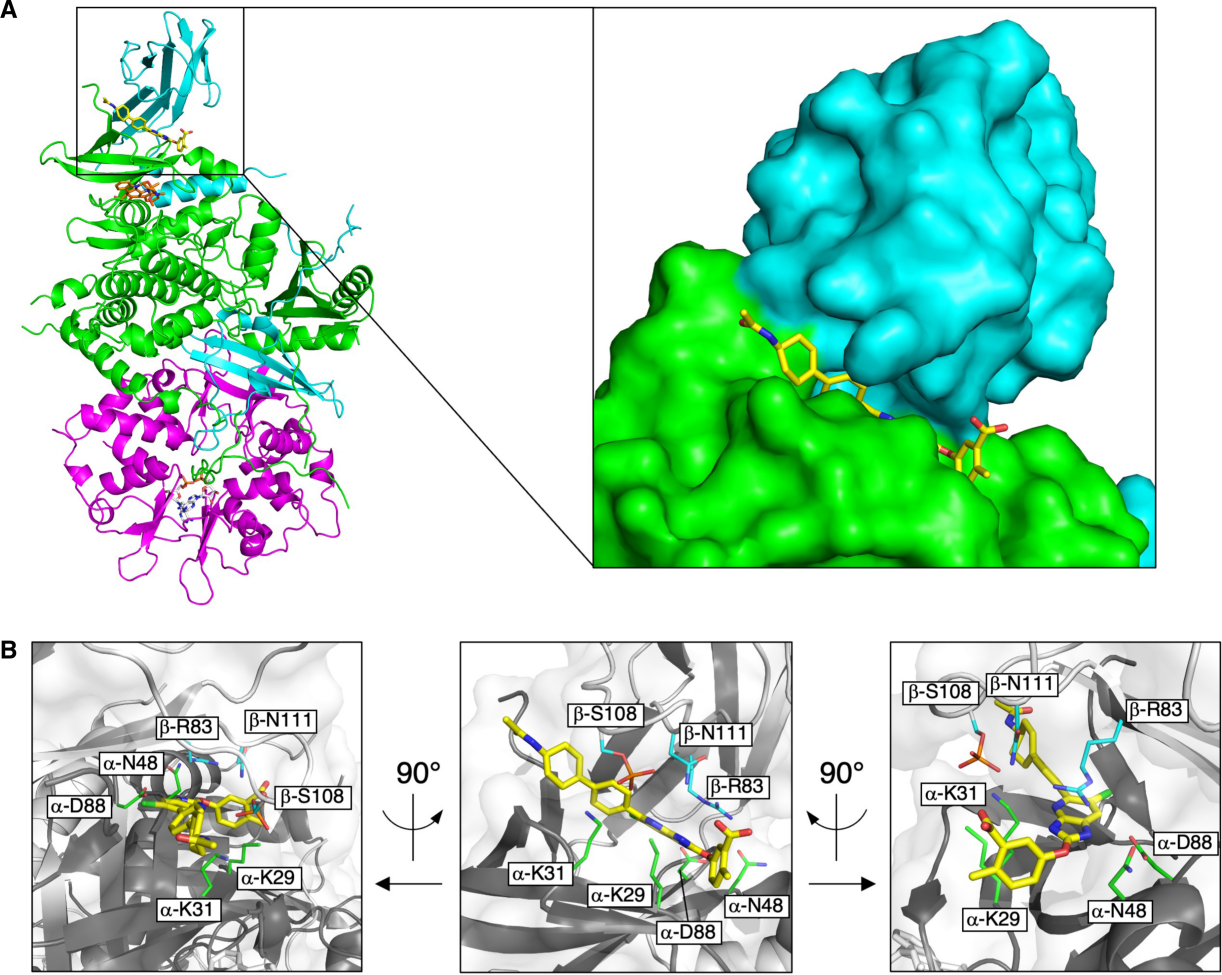
Crystal structure of MSG011 complexed with α2β1γ1. (**A**) Cartoon representation of α2β1γ1 (α2, green; β1, cyan; γ1, magenta) in complex with MSG011 (yellow), staurosporine (orange), and two AMP molecules (white). Inset: zoomed view of MSG011 bound to the ADaM site shown as a surface representation. (**B**) Close up views of the ADaM site with critical polar residues that interact with MSG011 shown as sticks, α2 residues are in green and β1 residues are in cyan. For clarity the cartoon representation of α2β1γ1 is shown in grey (α2, dark grey; β1, light grey) with a transparent surface view. Left and right images are 90° rotations of the middle image.

To structurally characterise the MSG011/AMPK interaction and investigate chemistry for blocking β1 binding we solved the co-crystal structure of activated α2β1γ1 (phosphorylated on α2-Thr172 and β1-Ser108) in complex with MSG011 at a resolution of 2.95 Å (Supplementary Table S1). As expected, MSG011 was docked in the ADaM site (Figure 4A and Supplementary Figure S6A) where clear electron density was observed for the drug in both AMPK heterotrimers in the asymmetric unit (Supplementary Figure S6B,C). Both MSG011 molecules were built in similar poses, however, missing electron density around the 2-methylbenzoic acid indicates flexibility in this region (Supplementary Figure S6A–C). This was also a feature of the SC4/AMPK complex structure [21]. Interestingly, α2-Lys29 is in close proximity to this ring and was unable to be completely modelled due to poor electron density around the ε-amino group (Figure 4B and Supplementary Figure S6A), hence it is plausible that it is participating in both hydrophobic interactions with the 2-methylbenzoic acid of MSG011 through the hydrophobic carbon tail, and hydrogen bonding with phosphorylated β1-Ser108 through its positively charged ε-amino group. There is also flexibility around the acetamido group, which was modelled in different conformations in each heterotrimer (Supplementary Figure S6A–C). This chemical group is not present in previously published ADaM site-directed activators and occupies a novel space at the entrance of the ADaM site, however it does not appear to interact with any AMPK residues that are resolved in this structure (Figure 4B and Supplementary Figure S6D). MSG011 differs from the α2 selective SC4 only in the LHS terminal ring, with addition of an acetamido and loss of aromaticity and a 2-hydroxyl group. We previously showed that the SC4 2-hydroxyl forms hydrogen bonding with α2-Lys31 [21], suggesting that loss of this interaction confers MSG011 with the ability to activate all AMPK complexes, and conversely that the 2-hydroxyl group is important for α2 selectivity of ADaM site compounds. It is important to note that other ADaM site compounds (SC4, A-769662 and R739) also possess a 2-hydroxyl group in the LHS terminal ring and don't display α2 selectivity, however due to differences in various other components of these drugs the reason for lack of selectivity is uncertain. Given that the majority of ADaM site drugs dock in a similar way, it seems likely that subtle global changes around the ADaM site pocket, both proximal and distal to the binding location, may contribute to isoform specificity and potency.

## Discussion

Recent biochemical and genetic data strongly implicate skeletal muscle α2β2γ1 AMPK as the molecular target by which ADaM site drugs stimulate glucose uptake in these tissues. However, detailed structure–function analyses of molecular components underpinning isoform selectivity are lacking as most series reported in the patent literature were solely assayed against α1β1γ1. Here, we show modification of the SC4 LHS phenyl group contributes to α isoform selectivity, although it remains unclear whether the SC4 2-hydroxylphenyl is a negative determinant for α1 activation, or the MSG010 and MSG011 substitutions are positive determinants. Other pan activators MK-8722, PF-739 and I-3-40 do not possess the 2-hydroxy modification of the biphenyl LHS fragment, although they all possess D-mannitol groups in place of the MSG011 2-methylbenzoic acid RHS moiety, which may also contribute to loss of isoform selectivity. Our structural analysis was unable to identify residues with the potential to interact with the MSG011 acetamide group, although it is worth noting that unresolved α and β N-terminal residues are likely in close proximity and may contribute to the ADaM binding pocket in manners specific to α/β isoform combinations. This could also explain why SC4 activates some α1β1 complexes but not α1β2 complexes [21], whereas other contributions, in particular β1-Asn111 and β2-Asp111 are also known to act as key factors in modulating sensitivity of β1- and β2-containing AMPK complexes [31]. MSG011 is a pan AMPK activator that effectively stimulated AMPK signalling in a panel of primary, immortalised and cancer cells. In most cases, the extent of AMPK signalling induced by MSG011 was comparable to that induced by severe energy stress. In hepatocytes, HEK293T and PC3 cells, MSG011-induced AMPK signalling was not accompanied by significant increases in phosphorylation of α-Thr172, nor perturbed adenylate nucleotide ratios in HEK293T cells. Thus, MSG011 acts primarily by allosterically enhancing intrinsic AMPK activity rather than by inducing energy stress or protecting phosphorylated α-Thr172 from dephosphorylation, although the latter mechanism may play a minor role in some cell types. Further structure/function analyses of α2β2-selective AMPK activators are warranted to aid the development of novel treatment strategies for major human metabolic diseases.

We were surprised by the discrepancy between our calculated EC_50_ values for MK-8722 and those previously reported, although some difference in calculated activating potency must be expected given extensive inter-assay variation. Possible reasons are numerous and include the uncharacterised influence of affinity tags used to purify AMPK, substrate composition, assay conditions, kinase detection method and source of recombinant AMPK that influences regulatory post-translational modifications. In terms of the latter, AMPK activation by extended CaMKK2 treatment, common practice in high throughput screening platforms [17,18,35], generates preparations with supraphysiological autophosphorylation of β-Ser108 (>95% vs. <10% basal β-pSer108 stoichiometry from mammalian cells [36,37]). Phosphorylation of β1-Ser108 stabilises the ADaM site and is required for AMPK sensitisation to lower potency activators A-769662, MT47-100, salicylate, long-chain fatty acyl CoA esters and lusianthridin [12,15,16,20,35,37], where the loss of phosphorylation via exchange for Ala reduces activating potency of SC4 by ∼4-fold and 991 by 40-fold [20,21]. To our knowledge the influence of β1-S108 phosphorylation on AMPK activation kinetics by MK-8722 and PF-739 has not been reported, however a similar sensitising effect is likely for all high potency pan activators and consequently assays using highly activated material may be expected to output more potent kinetics than those using more physiologically relevant preparations. It remains largely unknown what influence other abundant phosphorylation sites (e.g. β1-Ser182, α2-Ser345) and modifications (e.g. β-subunit myristoylation, missing in AMPK prepared from *E. coli*) have on drug sensitisation.

Increasing evidence indicates that ADaM site-targeting activators may induce energy stress at high concentrations, leading to phosphorylation of α-Thr172 by canonical AMP-dependent mechanisms (most recently investigated by Sanders and colleagues [35]) and supporting our previous recommendation that α-pThr172 should not be used in isolation as a marker of AMPK drug bioactivity [37]. Elevated cellular α-pThr172 is often used to demonstrate on-target action of AMPK drugs and is explained mechanistically by a drug-induced conformational change that makes α-pThr172 resistant to dephosphorylation. Here, we found that 2.5 µM MK-8722 induced robust phosphorylation of α-Thr172 in HEK293T/17 cells expressing WT γ1, an effect diminished by ∼55% in cells expressing the AMP-insensitive γ1 mutant R299G. Since A-769662-induced protection of α-pThr172 to phosphatases is retained in the γ1-R299G mutant [38], the most likely explanation in WT-expressing cells is that elevated AMP, arising from mitochondrial toxicity with 2.5 µM MK-8722, accounted for the majority of drug-induced increase in α-Thr172 phosphorylation. However, it is not a simple process to accurately measure the extent of energy stress induced by AMPK drugs; detecting changes in mitochondrial function (e.g. Seahorse) or adenylate nucleotide ratios (e.g. LC–MS) requires elaborate platforms and, in our experience, can produce confounding results since AMPK activators themselves will likely increase the ability of the cell to buffer against loss of mitochondrial ATP production. Relying on the inherent biological property of AMPK to respond to even small fluctuations in AMP/ADP/ATP ratios, a simpler option would be to routinely compare candidate AMPK activators in WT AMPK cell lines with their γ1-R299G stably expressing counterparts to reveal purely allosteric effects on signalling [15].

## Materials and methods

### General experimental details for organic synthesis

Unless stated specifically, all chemicals were purchased from commercial suppliers and used without purification. All reactions were conducted in oven-dried glassware under nitrogen atmosphere. Progress of reactions was tracked by TLC and was performed on silica gel 60 F254 aluminium sheets (0.25 mm, Merck). ^1^H (400.13 MHz) and ^13^C NMR (100.62 MHz) NMR spectra for each compound were collected from a Bruker Avance III Nanobay spectrometer with a BACS 60 sample changer using deuterated solvents from Cambridge Isotope Laboratories. Chemical shifts (δ, ppm) are reported relative to the solvent peak (CDCl_3_: 7.26 [^1^H] or 77.16 [^13^C]; DMSO-*d*_6_: 2.50 [^1^H] or 39.52 [^13^C]; MeOD: 3.31 [^1^H] or 49.00 [^13^C]). Coupling constants are reported in hertz (Hz), and the following abbreviations are used to assign the multiplicity of the ^1^H NMR signal: s = singlet; bs = broad singlet; d = doublet; t = triplet; q = quartet; quin = quintet; dd = doublet of doublets; m = multiplet. Analytical HPLC was performed on an Agilent 1260 Infinity analytical HPLC coupled with a G1322A degasser, G1312B binary pump, G1367E high-performance autosampler, and G4212B diode array detector. Conditions were as follows: Zorbax Eclipse Plus C18 rapid resolution column (4.6 × 100 mm) with UV detection at 254 and 214 nm, 30°C; the sample was eluted using a gradient system, where solvent A was 0.1% aq. TFA and solvent B was 0.1% TFA in MeCN (5−100% B [9 min], 100% B [1 min]; 0.5 ml/min). High-resolution mass spectra were acquired on an Agilent 6224 TOF LCMS coupled to an Agilent 1290 Infinity LC. All data were acquired and reference mass corrected via a dual-spray electrospray ionisation (ESI) source. Each scan or data point on the total ion chromatogram (TIC) is an average of 13 700 transients, producing a spectrum every second. Mass spectra were created by averaging the scans across each peak and subtracting the background from first 10 sec of the TIC. Acquisition was performed using the Agilent Mass Hunter Data Acquisition software (ver. B.05.00, build 5.0.5042.2), and analysis was performed using Mass Hunter Qualitative Analysis (ver. B.05.00 build 5.0.519.13). Acquisition parameters were as follows: mode, ESI; drying gas flow, 11 L/min; nebulizer pressure, 45 psi; drying gas temperature, 325°C; voltages: capillary, 4000 V; fragmentor, 160 V; skimmer, 65 V; octapole RF, 750 V; scan range, 100−1500 m/z; and positive ion mode internal reference ions, m/z 121.050873 and 922.009798. LC conditions were as follows: Agilent Zorbax SB-C18 rapid resolution HT (2.1 × 50 mm, 1.8 µm column), 30°C; the sample (5 µl) was eluted using a binary gradient (solvent A: 0.1% aq. HCO_2_H; solvent B: 0.1% HCO_2_H in MeCN; 5−100% B [3.5 min], 0.5 ml/min).

#### Procedures for the preparation of MSG008 and MSG009 and their intermediates

##### Methyl 5-((6-chloro-5-iodo-1-((2-(trimethylsilyl)ethoxy)methyl)-1H-imidazo[4,5-b]pyridin-2-yl)oxy)-2-methylbenzoate (1)

The title compound was prepared using the literature procedure [21], and it was obtained as a colourless oil (783 mg, 1.36 mmol) in 83% yield. HRMS (ESI) m/z: [M + H]^+^ Calcd for C_21_H_25_ClIN_3_O_4_Si 574.0426; Found 574.0431.

##### 2-(3-(2-Methoxyethyl)phenyl)-4,4,5,5-tetramethyl-1,3,2-dioxaborolane

1-Bromo-3-(2-methoxyethyl)benzene [39] (882 mg, 4.10 mmol, 1 equiv), potassium acetate (1.21 g, 12.3 mmol, 3.0 equiv), B_2_pin_2_ (1.53 g, 6.03 mmol, 1.5 equiv) and Pd(dppf)Cl_2_·CH_2_Cl_2_ (165 mg, 202 µmol, 5 mol%) were added to a round bottom flask then evacuated and back-filled with nitrogen gas three times. DMF (9.00 ml) was bubbled with nitrogen gas for 10 min before added to the substrates. The reaction mixture was heated at 120°C overnight then concentrated under reduced pressure. The dried residue was dissolved in ethyl acetate (10.0 ml) and filtered through filter aid. The filter aid cake was washed with ethyl acetate (10.0 ml × 5). The collected filtrate was washed with saturated sodium bicarbonate solution (25.0 ml), water (25.0 ml) then brine (25.0 ml). The filtrate was then dried over magnesium sulfate and concentrated under reduced pressure. After column chromatography (8–15% ethyl acetate in petroleum ether), the title compound was collected as a pale green oil (623 mg, 2.38 mmol) in 58% yield. ^1^H NMR (400 MHz, CDCl_3_) δ 7.67–7.66 (m, 2H), 7.35–7.28 (m, 2H), 3.61 (t, *J* = 7.2 Hz, 2H), 3.36 (s, 3H), 2.90 (t, *J* = 7.2 Hz, 2H), 1.35 (s, 12H) ppm. ^13^C NMR (101 MHz, CDCl_3_) δ 138.3, 135.3, 132.9, 132.0, 128.0, 83.9, 73.9, 58.8, 36.3, 25.0 ppm. HRMS (ESI) m/z: [M + H]^+^ Calcd for C_15_H_23_BO_3_ 263.1816; Found 263.1822.

##### 5-((6-Chloro-5-(3-(2-methoxyethyl)phenyl)-1-((2-(trimethylsilyl)ethoxy)methyl)-1H-imidazo[4,5-b]pyridin-2-yl)oxy)-2-methylbenzoate (2a)

Methyl 5-((6-chloro-5-iodo-1-((2-(trimethylsilyl)ethoxy)methyl)-1*H*-imidazo[4,5-*b*]pyridin-2-yl)oxy)-2-methylbenzoate 1 (195 mg, 0.340 mmol, 1 equiv), 2-(3-(2-methoxyethyl)phenyl)-4,4,5,5-tetramethyl-1,3,2-dioxaborolane (116 mg, 0.441 mmol, 1.3 equiv), Cs_2_CO_3_ (181 mg, 0.556 mmol, 1.6 equiv) and Pd(dppf)Cl_2_ (31.2 mg, 42.6 µmol, 12.5 mol%) were added to a round bottom flask then evacuated and back-filled with nitrogen gas three times. A solvent mixture of DMF (9.00 ml) and water (1.00 ml) was bubbled with nitrogen gas for 10 min before adding it to the substrates. The reaction mixture was heated at 80°C overnight then concentrated under reduced pressure and partitioned between water (10.0 ml) and ethyl acetate (15.0 ml). The aqueous fraction was isolated and extracted with ethyl acetate (15.0 ml × 2). The combined organic fractions were washed with brine (10.0 ml), dried over sodium sulfate and concentrated under reduced pressure. After column chromatography (15–25% ethyl acetate in petroleum benzine), the title compound was collected as colourless oil (114 mg, 0.195 mmol) in 58% yield. ^1^H NMR (400 MHz, CDCl_3_) δ 7.96 (d, *J* = 2.8 Hz, 1H), 7.87 (s, 1H), 7.59–7.56 (m, 2H), 7.48 (dd, *J* = 8.4, 2.8 Hz, 1H), 7.41–7.35 (m, 2H), 7.31–7.28 (m, 1H), 5.67 (s, 2H), 3.89 (s, 3H), 3.82–3.78 (m, 2H), 3.66 (t, *J* = 7.2 Hz, 2H), 3.38 (s, 3H), 2.97 (t, *J* = 7.2 Hz, 2H), 2.64 (s, 3H), 1.02–0.97 (m, 2H), −0.03 (s, 9H) ppm. ^13^C NMR (101 MHz, CDCl_3_) δ 166.9, 158.0, 150.5, 149.6, 145.4, 139.0, 138.8, 138.6, 133.3, 132.4, 130.9, 130.4, 129.1, 128.1, 127.9, 127.2, 124.7, 124.4, 122.9, 73.8, 70.1, 67.8, 58.8, 52.2, 36.4, 21.4, 18.1, −1.3 ppm. HRMS (ESI) m/z: [M + H]^+^ Calcd for C_30_H_36_ClN_3_O_5_Si 582.2186; Found 582.2200.

##### 5-((6-Chloro-5-(3-(2-methoxyethyl)phenyl)-1H-imidazo[4,5-b]pyridin-2-yl)oxy)-2-methylbenzoic acid (MSG008)

Formic acid (4.00 ml, 106 mmol, 530 equiv) and saturated KHSO_4_ solution (0.250 ml) was added to methyl 5-((6-chloro-5-(3-(2-methoxyethyl)phenyl)-1-((2-(trimethylsilyl)ethoxy)methyl)-1*H*-imidazo[4,5-*b*]pyridin-2-yl)oxy)-2-methylbenzoate 2a (114 mg, 0.195 mmol, 1 equiv). The reaction solution was heated at 80°C for 1 h before partitioned between water (12.0 ml) and ethyl acetate (24.0 ml). The aqueous fraction was isolated and extracted with ethyl acetate (12.0 ml). The combined organic fractions were washed with brine (20.0 ml), dried over sodium sulfate and concentrated under reduced pressure. The dried residue was then dissolved in methanol (9.00 ml) and treated with 2 N KOH (0.980 ml, 1.95 mmol, 10 equiv). The reaction solution was heated at 80°C overnight before solvent removal under reduced pressure. The residue was acidified with 10% citric acid solution (10.0 ml) and extracted with ethyl acetate (15.0 ml × 2). The combined organic fractions were washed with brine (10.0 ml), dried over sodium sulfate and concentrated under reduced pressure. After column chromatography (5–15% methanol in dichloromethane), the title compound was collected as a white solid (35.4 mg, 80.8 µmol) in 41% yield. ^1^H NMR (400 MHz, MeOD) δ 7.90 (d, *J* = 2.8 Hz, 1H), 7.84 (s, 1H), 7.41–7.35 (m, 2H), 7.30–7.28 (m, 1H), 3.65 (t, *J* = 6.8 Hz, 2H), 3.34 (s, 3H), 2.93 (t, *J* = 6.8 Hz, 2H), 2.62 (s, 3H) ppm. ^13^C NMR (101 MHz, MeOD) δ 169.9, 161.0, 152.0, 150.9, 140.1, 140.0, 139.1, 134.2, 133.5, 133.1, 131.3, 129.9, 128.9, 128.6, 125.0, 124.7, 123.6, 120.1, 118.1, 74.6, 58.8, 37.0, 21.3 ppm. HRMS (ESI) m/z: [M + H]^+^ Calcd for C_23_H_20_ClN_3_O_4_ 438.1215; Found 438.1227.

##### Methyl 5-((6-chloro-5-((2-hydroxyphenyl)ethynyl)-1-((2-(trimethylsilyl)ethoxy)methyl)-1H-imidazo[4,5-b]pyridin-2-yl)oxy)-2-methylbenzoate (2b)

Methyl 5-((6-chloro-5-iodo-1-((2-(trimethylsilyl)ethoxy)methyl)-1*H*-imidazo[4,5-*b*]pyridin-2-yl)oxy)-2-methylbenzoate 1 (375 mg, 0.654 mmol), 2-ethynylphenol (225 mg, 1.90 mmol, 2.9 equiv), Pd(PPh_3_)Cl_2_ (25.8 mg, 36.8 µmol, 6 mol%) and CuI (19.8 mg, 0.104 mmol, 16 mol%) were added to a round bottom flask, evacuated and back-filled with nitrogen gas three times. A solvent mixture of THF (10.0 ml) and diisopropylamine (3.50 ml) was bubbled with nitrogen gas for 10 min before adding to the substrates. The reaction mixture was left stirring at rt overnight before saturated ammonium chloride solution (20.0 ml) was added. The aqueous fraction was isolated and extracted with diethyl ether (25.0 ml × 2). The combined organic fractions were washed with brine (15.0 ml), dried over sodium sulfate and concentrated under reduced pressure. After column chromatography (15–35% ethyl acetate in petroleum benzine), the title compound was collected as a yellow solid (306 mg, 0.543 mmol) in 83% yield. ^1^H NMR (400 MHz, CDCl_3_) δ 7.95 (d, *J* = 2.8 Hz, 1H), 7.84 (s, 1H), 7.52 (dd, *J* = 7.6, 1.6 Hz, 1H), 7.46 (dd, *J* = 8.4, 2.8 Hz, 1H), 7.36 (d, *J* = 8.4 Hz, 1H), 7.34–7.30 (m, 1H), 7.01 (dd, *J* = 8.4, 0.8 Hz, 1H). 6.94 (td, *J* = 7.6, 1.2 Hz, 1H), 5.68 (s, 2H), 3.90 (s, 3H), 3.82–3.78 (m, 2H), 2.64 (s, 3H), 1.04–0.99 (m, 2H), 0.00 (s, 9H) ppm. ^13^C NMR (101 MHz, CDCl_3_) δ 166.8, 158.8, 157.8, 150.3, 145.6, 139.1, 133.7, 133.4, 133.2, 131.8, 131.5, 131.0, 128.9, 126.0, 124.4, 123.0, 120.6, 115.2, 108.9, 93.8, 88.4, 70.2, 67.9, 52.2, 21.4, 18.0, −1.3 ppm. HRMS (ESI) m/z: [M + H]^+^ Calcd for C_29_H_30_ClN_3_O_5_Si 564.1716; Found 564.1733.

##### 5-((6-Chloro-5-((2-hydroxyphenyl)ethynyl)-1H-imidazo[4,5-b]pyridin-2-yl)oxy)-2-methylbenzoic acid (MSG009)

Formic acid (6.00 ml, 159 mmol, 306 equiv) and saturated KHSO_4_ solution (0.610 ml) was added to methyl 5-((6-chloro-5-((2-hydroxyphenyl)ethynyl)-1-((2-(trimethylsilyl)ethoxy)methyl)-1*H*-imidazo[4,5-*b*]pyridin-2-yl)oxy)-2-methylbenzoate 2b (292 mg, 518 µmol, 1 equiv). The reaction solution was heated at 80°C for 1.5 h before partitioned between brine (50.0 ml) and ethyl acetate (50.0 ml). The aqueous fraction was further extracted with ethyl acetate (25.0 ml × 2). The combined organic fractions were washed with brine (30.0 ml), dried over sodium sulfate and concentrated under reduced pressure. The dried residue was then dissolved in methanol (24.0 ml) and 2 N KOH (2.60 ml, 5.20 mmol, 10 equiv) was added. The solution was heated at 80°C overnight, concentrated under reduced pressure, acidified with 10% citric acid aqueous solution (20.0 ml) and extracted with ethyl acetate (30.0 ml). The aqueous fraction was isolated and further extracted with ethyl acetate (15.0 ml × 2). The combined organic fractions were washed with brine (30.0 ml), dried over sodium sulfate and concentrated under reduced pressure. After column chromatography (5–15% methanol in dichloromethane) and a subsequent preparative HPLC (40–85% acetonitrile in water with 0.1% TFA), the title compound was obtained as a light green solid (14.9 mg, 35.5 µmol) in 7% yield. ^1^H NMR (400 MHz, MeOD) δ 7.92 (d, *J* = 2.8 Hz, 1H), 7.83 (s, 1H), 7.48 (dd, *J* = 8.4, 2.8 Hz, 1H), 7.43 (dd, *J* = 7.6, 1.6 Hz, 1H), 7.39 (d, *J* = 8.4 Hz, 1H), 7.27–7.23 (m, 1H), 6.89–6.83 (m, 2H), 2.61 (s, 3H) ppm. ^13^C NMR (101 MHz, MeOD) δ 169.7, 161.9, 160.0, 151.9, 145.7, 139.5, 134.9, 134.3, 134.2, 132.9, 132.2, 132.0, 128.8, 125.2, 123.9, 123.7, 120.7, 116.8, 110.5, 91.7, 91.0, 21.3 ppm. HRMS (ESI) m/z: [M + H]^+^ Calcd for C_22_H_14_ClN_3_O_4_ 420.0746; Found 420.0749.

#### Procedures for the preparation of MSG010

##### 4′-(2-(3-carboxy-4-methylphenoxy)-6-chloro-1H-imidazo[4,5-b]pyridin-5-yl)-2,3,4,5-tetrahydro-[1,1′-biphenyl]-4-carboxylic acid (MSG010)

To a solution of ethyl 5-hydroxy-2-methylbenzoate (108 mg, 0.600 mmol, 1 equiv) in DMF (6.00 ml) was added Cs_2_CO_3_ (488 mg, 1.50 mmol, 2.5 equiv) and 6-chloro-5-iodo-2-(methylsulfonyl)-1-((2-(trimethylsilyl)ethoxy)methyl)-1H-imidazo[4,5-b]pyridine 3 (292 mg, 0.600 mmol, 1 equiv). The reaction was stirred at room temperature for 2 h. The solvent was removed *in vacuo*, the residue was acidified with a 10% aq. citric acid solution then extracted with EtOAc (×3). The combined organic extracts were washed with water, brine, then dried (MgSO_4_) and concentrated *in vacuo* to give the crude material. The product was purified by silica gel chromatography (0–30% EtOAc/n-Hexane) to afford intermediate 4 (0.200 g, 0.340 mmol) in 57% yield. ESI-MS: m/z = 588.0 [M + H]^+^.

[1,1′-Bis(diphenylphosphino)ferrocene]dichloropalladium(II) (25.0 mg, 0.0340 mmol, 10 mol%) was added to a DMF (9.00 ml)/water (1.00 ml) solution of intermediate 4 (0.200 g, 0.340 mmol, 1 equiv), ethyl 4′-(4,4,5,5-tetramethyl-1,3,2-dioxaborolan-2-yl)-2,3,4,5-tetrahydro-[1,1′-biphenyl]-4-carboxylate (145 mg, 0.408 mmol, 1.2 equiv) and caesium carbonate (166 mg, 0.510 mmol, 1.5 equiv) at rt under a nitrogen atmosphere, the reaction was heated to 80°C for 3 h. The reaction was cooled to room temperature, concentrated, and then partitioned between water and EtOAc. The aqueous layer was extracted with EtOAc (30.0 ml × 2) and the combined organic extracts were washed with brine, dried over MgSO_4_ and the solvent was removed *in vacuo* to give the crude material. The product was purified by silica gel chromatography (0–40% EtOAc/n-Hexane) to afford intermediate 5 (120 mg, 0.174 mmol) in 51% yield. ESI-MS: m/z = 690.2 [M + H]^+^.

TBAF (1.0 M in THF) (3.00 ml, 3.00 mmol, 17 equiv) was added to a solution of intermediate 5 (120 mg, 0.174 mmol, 1 equiv) in THF (3.00 ml). The reaction was heated at 80°C for 3 h. The volatiles were removed *in vacuo* and the resultant residue dissolved in MeOH (10.0 ml) and a 2.5 M aq. sol. of NaOH (4.00 ml, 10.0 mmol, 57 equiv). The solution was stirred at room temperature for 8 h. Subsequently the volatiles were removed *in vacuo*, the residue was taken up in water (10.0 ml) and adjusted to pH 7 with 2 M aq. sol. of HCl, then extracted with EtOAc. The combined organic extracts were washed with brine, dried over MgSO_4_ and the solvent removed *in vacuo*. The resultant crude material was purified by silica gel chromatography (0–20% CH_3_OH/CH_2_Cl_2_) to afford MSG010 (30.0 mg, 59.5 µmol) as an off-white solid in 34% yield. ^1^H NMR (400 MHz, DMSO) δ 7.90 (s, 1H), 7.69 (bs, 1H), 7.58 (d, *J* = 8.0 Hz, 2H), 7.48 (d, *J* = 8.0 Hz, 2H), 7.39 (d, *J* = 7.2 Hz, 1H), 7.32 (d, *J* = 8.4 Hz, 1H), 6.24 (bs, 1H), 2.50–2.30 (m, 8H), 2.09–2.07 (m, 1H), 1.75–1.65 (m, 1H) ppm. ^13^C NMR (101 MHz, DMSO) δ 176.8, 169.0, 160.4, 150.8, 149.0, 147.6, 140.7, 137.5, 135.6, 135.2, 135.0, 132.5, 131.3, 129.6, 124.2, 123.6, 123.4, 122.7, 121.9, 121.6, 38.3, 28.1, 26.0, 25.4, 20.6 ppm. HRMS (ESI) m/z: [M + H]^+^ Calcd for C_27_H_22_ClN_3_O_5_ 504.1321; Found 504.1336.

#### Procedures for the preparation of MSG011 and its intermediates

##### tert-Butyl (4′-bromo-2,3,4,5-tetrahydro-[1,1′-biphenyl]-4-yl)carbamate (7)

A suspension of 4-(*N*-Boc-amino)cyclohex-1-enyl-1-boronic acid pinacol ester 6 (3.00 g, 9.27 mmol, 1 equiv), 1-bromo-4-iodobenzene (3.94 g, 13.9 mmol, 1.5 equiv), Pd(dppf)Cl_2_ (673 mg, 0.920 mmol, 0.1 equiv), caesium carbonate (9.07 g, 27.9 mmol, 3 equiv) in dioxane/H_2_O (10:1, 27.5 ml) was heated at 90°C overnight. The suspension was then filtered and concentrated under reduced pressure. The residue was partitioned between ethyl acetate (80.0 ml) and brine (80.0 ml). The organic fraction was isolated and the aqueous layer was extracted with ethyl acetate (3 × 30.0 ml). The combined organic fractions were washed with brine (30.0 ml) then dried over magnesium sulfate. After column chromatography (20–40% ethyl acetate/petroleum benzine), the title compound was collected as a white solid (2.24 g, 6.36 mmol) in 69% yield. ^1^H NMR (400 MHz, CDCl_3_) δ 7.42 (d, *J* = 8.8 Hz, 2H), 7.23 (d, *J* = 8.4 Hz, 2H), 6.02–6.00 (m, 1H), 4.55 (bs, 1H), 3.85 (bs, 1H), 2.60–2.49 (m, 3H), 2.09–2.01 (m, 2H), 1.76–1.67 (m, 1H), 1.46 (s, 9H) ppm. ^13^C NMR (101 MHz, CDCl_3_) δ 155.5, 140.5, 135.6, 131.4, 126.8, 122.6, 120.9, 79.4, 45.4, 32.8, 28.8, 28.6, 25.7 ppm. HRMS (ESI) m/z: [M + Na]^+^ Calcd for C_17_H_22_BrNO_2_ 374.0726; Found 374.0702.

##### 4′-Bromo-2,3,4,5-tetrahydro-[1,1′-biphenyl]-4-amine trifluoroacetate (8)

At 0°C, trifluoroacetic acid (7.00 ml, 10.4 g, 91.4 mmol, 22 equiv) was added to a suspension of *tert*-butyl (4′-bromo-2,3,4,5-tetrahydro-[1,1′-biphenyl]-4-yl)carbamate 7 (1.50 g, 4.25 mmol, 1 equiv) in dichloromethane (10.0 ml). The suspension turned into clear brown solution and it was stirred at rt for 2.5 h. The reaction solution was concentrated under reduced pressure to give a solid residue. After recrystallisation in cyclohexane, the title compound was collected as beige solid (1.55 g, 4.23 mmol) in quantitative yield. ^1^H NMR (400 MHz, MeOD) δ 7.45 (d, *J* = 8.4 Hz, 2H), 7.32 (d, *J* = 8.4 Hz, 2H), 6.07–6.05 (m, 1H), 3.48–3.41 (m, 1H), 2.69–2.58 (m, 3H), 2.33–2.16 (m, 2H), 1.91–1.81 (m, 1H) ppm. ^13^C NMR (101 MHz, MeOD) δ 141.3, 137.2, 132.4, 128.0, 122.0, 121.4, 47.9, 31.0, 27.9, 26.5 ppm. HRMS (ESI) m/z: [M + H]^+^ Calcd for C_12_H_14_BrN 252.0382; Found 252.0386.

##### N-(4′-Bromo-2,3,4,5-tetrahydro-[1,1′-biphenyl]-4-yl)acetamide (9)

Acetic anhydride (4.50 ml, 4.86 g, 47.6 mmol, 17 equiv) was added to a solution of 4′-bromo-2,3,4,5-tetrahydro-[1,1′-biphenyl]-4-amine trifluoroacetate 8 (1.00 g, 2.73 mmol, 1 equiv) in pyridine (4.50 ml, 4.40 g, 55.6 mmol, 20 equiv) at room temperature. White precipitate was formed shortly after the addition and the suspension was heated at 80°C for 4 h before concentrating under reduced pressure. The residue was suspended in ethyl acetate (30.0 ml) and washed with saturated sodium bicarbonate solution (20.0 ml), water (20.0 ml) and brine (20.0 ml). The organic fraction was dried over magnesium sulfate and concentrated under reduced pressure. After recrystallisation in cyclohexane, the title compound was collected as a beige solid (483 mg, 1.64 mmol) in 60% yield. ^1^H NMR (400 MHz, MeOD) δ 7.43 (d, *J* = 8.8 Hz, 2H), 7.31 (d, *J* = 8.4 Hz, 2H), 6.08–6.06 (m, 1H), 4.01–3.94 (m, 1H), 2.54–2.47 (m, 3H), 2.15–1.97 (m, 2H), 1.95 (s, 3H), 1.75–1.66 (m, 1H) ppm. ^13^C NMR (101 MHz, MeOD) δ 172.8, 142.1, 136.8, 132.3, 127.9, 123.7, 121.6, 46.1, 32.8, 29.5, 27.2, 22.7 ppm. HRMS (ESI) m/z: [M + H]^+^ Calcd for C_14_H_16_BrNO 294.0488; Found 294.0493.

##### N-(4′-(4,4,5,5-Tetramethyl-1,3,2-dioxaborolan-2-yl)-2,3,4,5-tetrahydro-[1,1′-biphenyl]-4-yl)acetamide (10)

A suspension of *N*-(4′-bromo-2,3,4,5-tetrahydro-[1,1′-biphenyl]-4-yl)acetamide 9 (482 mg, 1.64 mmol, 1 equiv), potassium acetate (483 mg, 4.92 mmol, 3 equiv), B_2_pin_2_ (615 mg, 2.42 mmol, 1.5 equiv) and Pd(dppf)Cl_2_•CH_2_Cl_2_ (69.1 mg, 84.6 µmol, 5 mol%) in DMF (6.00 ml) was heated overnight at 120°C. The suspension was concentrated under reduced pressure and the residue was suspended in ethyl acetate (10.0 ml) and filtered through a plug of Celite. The Celite plug was then washed with ethyl acetate (6 × 10.0 ml). The combined organic filtrate was washed with saturated NaHCO_3_ solution (25.0 ml), water (25.0 ml) and brine (25.0 ml) The organic fraction was dried over sodium sulfate and concentrated under reduced pressure. After column chromatography (4% methanol/dichloromethane), the title compound was collected as pale brown oil (297 mg, 0.869 mmol) in 53% yield. ^1^H NMR (400 MHz, CDCl_3_) δ 7.76 (d, *J* = 8.4 Hz, 2H), 7.38 (d, *J* = 8.4 Hz, 2H), 6.11–6.09 (m, 1H), 5.51–5.49 (m, 1H), 4.24–4.16 (m, 1H), 2.63–2.47 (m, 3H), 2.11–2.02 (m, 2H), 1.99 (s, 3H), 1.83–1.73 (m, 1H), 1.34 (s, 12H) ppm. ^13^C NMR (101 MHz, CDCl_3_) δ 169.7, 144.2, 136.7, 135.0, 124.4, 122.6, 83.9, 44.3, 32.5, 28.4, 25.5, 25.0, 23.8 ppm. HRMS (ESI) m/z: [M + H]^+^ Calcd for C_20_H_28_BNO_3_ 342.2239; Found 342.2246.

##### Methyl 5-((5-(4′-acetamido-2′,3′,4′,5′-tetrahydro-[1,1′-biphenyl]-4-yl)-6-chloro-1-((2-(trimethylsilyl)ethoxy)methyl)-1H-imidazo[4,5-b]pyridin-2-yl)oxy)-2-methylbenzoate (11)

A suspension of compound 10 (293 mg, 0.858 mmol, 1.1 equiv), compound 1 (436 mg, 0.760 mmol, 1 equiv), Cs_2_CO_3_ (348 mg, 1.07 mmol, 1.4 equiv) and Pd(dppf)Cl_2_ (53.6 mg, 73.2 µmol, 10 mol%) in DMF (18.0 ml) and water (2.00 ml) was heated at 80°C for 4 h 45 min. The suspension was then concentrated under reduced pressure. The residue was suspended in water (20.0 ml) and extracted with ethyl acetate (3 × 15.0 ml). The combined organic fractions were washed with brine (25.0 ml) and dried over magnesium sulfate. After column chromatography (10% petroleum benzine/ethyl acetate), the title compound was collected as white solid (221.7 mg, 0.335 mmol) in 44% yield. ^1^H NMR (400 MHz, CDCl_3_) δ 7.95 (d, *J* = 2.4 Hz, 1H), 7.86 (s, 1H), 7.73–7.71 (m, 2H), 7.48–7.46 (m, 3H), 7.37–7.35 (m, 1H), 6.13–6.12 (m, 1H), 5.66 (s, 2H), 5.57–5.56 (m, 1H), 4.26–4.18 (m, 1H), 3.89 (s, 3H), 3.82–3.78 (m, 2H), 2.64–2.59 (m, 6H), 2.00 (s, 3H), 1.84–1.71 (m, 2H), 1.01–0.97 (m, 2H), −0.03 (s, 9H) ppm. ^13^C NMR (101 MHz, CDCl_3_) δ 169.7, 166.9, 158.0, 150.4, 149.0, 145.5, 141.4, 138.8, 137.6, 136.3, 133.3, 132.4, 130.8, 129.9, 127.2, 124.6, 124.5, 123.0, 122.3, 70.0, 67.8, 52.2, 44.4, 32.5, 28.4, 25.6, 23.8, 21.4, 18.0, −1.3 ppm. (One quaternary aromatic carbon was not detected possibly due to overlapping with another quaternary aromatic carbon) HRMS (ESI) m/z: [M + H]^+^ Calcd for C_35_H_41_ClN_4_O_5_Si 661.2608; Found 661.262.

##### 5-((5-(4′-Acetamido-2′,3′,4′,5′-tetrahydro-[1,1′-biphenyl]-4-yl)-6-chloro-1H-imidazo[4,5-b]pyridin-2-yl)oxy)-2-methylbenzoic acid (MSG011)

To a solution of compound 11 (118 mg, 179 µmol, 1 equiv) in formic acid (2.00 ml) was added saturated KHSO_4_ aqueous solution (210 µl). The solution was heated at 80°C for 1.5 h before it was partitioned between water (15.0 ml) and ethyl acetate (30.0 ml). The organic fraction was then washed with brine (10.0 ml) and concentrated under reduced pressure to give a white solid. The white solid was suspended in methanol (8.50 ml), treated with 2 N KOH (0.890 ml, 1.78 mmol, 10 equiv) and heated to 80°C for 4.5 h. The reaction solution was concentrated under reduced pressure and the residue was added to 10% citric acid (10.0 ml) and ethyl acetate (5.00 ml). Off-white solid precipitate was observed, collected through filtration and washed with a mixture of water (2.00 ml) and ethyl acetate (5.00 ml). The titled compound was collected as an off-white solid (62.0 mg, 120 µmol) in 68% yield (HPLC purity >95%). ^1^H NMR (400 MHz, DMSO) δ 7.97 (s, 1H), 7.86 (d, *J* = 7.6 Hz, 1H), 7.82 (d, *J* = 2.8 Hz, 1H), 7.62–7.60 (m, 2H), 7.53–7.50 (m, 3H), 7.42–7.40 (m, 1H), 6.18 (bs, 1H), 3.90–3.81 (m, 1H), 2.55 (s, 3H), 2.51–2.47 (m, 3H), 2.12–2.04 (m, 1H), 1.94–1.90 (m, 1H), 1.82 (s, 3H), 1.66–1.57 (m, 1H) ppm. ^13^C NMR (101 MHz, DMSO) δ 177.0, 171.7, 169.4, 168.1, 159.7, 150.7, 148.1, 140.8, 137.3, 136.8, 135.3, 133.1, 132.3, 129.7 (2C), 124.4, 124.0, 123.1, 122.4, 122.1, 44.2, 31.9, 28.5, 25.9, 22.9, 20.8 ppm. HRMS (ESI) m/z: [M + H]^+^ Calcd for C_28_H_25_ClN_4_O_4_ 517.1637; Found 517.1648.

#### Procedures for the preparation of MSG012

##### 4′-(6-Chloro-2-(((1s,4s)-4-(dimethylcarbamoyl)-4-hydroxycyclohexyl)oxy)-1H-imidazo[4,5-b]pyridin-5-yl)-2,3,4,5-tetrahydro-[1,1′-biphenyl]-4-carboxylic acid (MSG012)

[1,1′-Bis(diphenylphosphino)ferrocene]dichloropalladium(II) (73.0 mg, 0.100 mmol, 10 mol%) was added to a DMF(9.00 ml)/water(1.00 ml) solution of 6-chloro-5-iodo-2-(methylsulfonyl)-1H-imidazo[4,5-b]pyridine 3 (357 mg, 1.00 mmol, 1 equiv), ethyl 4′-(4,4, 5,5-tetramethyl-1,3,2- dioxaborolan-2-yl)-2,3,4,5-tetrahydro-[1,1′-biphenyl]-4-carboxylate (392 mg, 1.10 mmol, 1.1 equiv) and caesium carbonate (488 mg, 1.50 mmol, 1.5 equiv) at rt under nitrogen atmosphere, the reaction was heated to 80°C for 3 h. The reaction was cooled to room temperature, concentrated, and then partitioned between water and EtOAc. The aqueous layer was extracted with EtOAc (30.0 ml × 2) and the combined organic extracts were washed with brine, dried over MgSO_4_ and the solvent removed *in vacuo* to give the crude material. The product was purified by silica gel chromatography (0-50% EtOAc/n-Hexane) to afford intermediate 12 (300 mg, 0.652 mmol) in 65% yield. ESI-MS: m/z = 460.1 [M + H]^+^.

To a 0°C solution of intermediate 12 (300 mg, 0.652 mmol, 1 equiv) in THF (20.0 ml) was added triethylamine (99 mg, 0.978 mmol, 1.5 equiv) and SEM-Cl (120 mg, 0.717 mmol, 1.1 equiv). The reaction was warmed to room temperature, concentrated, and then partitioned between water and EtOAc. The aqueous layer was extracted with EtOAc (30.0 ml × 2) and the combined organic phases washed with brine, dried over MgSO_4_ and the solvent removed *in vacuo* to give the crude material. The product was purified by silica gel chromatography (0–40% EtOAc/n-Hexane) to afford intermediate 13 as a light yellow solid (320 mg, 0.542 mmol) in 83% yield. ^1^H NMR (400 MHz, DMSO) δ 8.31 (s, 1H), 7.79–7.71 (m, 2H), 7.56–7.47 (m, 2H), 6.36–6.26 (m, 1H), 6.06 (s, 2H), 4.26–4.17 (m, 2H), 3.79–3.71 (m, 2H), 3.51 (s, 3H), 2.72–2.50 (m, 5H), 2.29–2.21 (m, 1H), 1.97–1.85 (m, 1H), 1.35–1.29 (m, 3H), 1.03–0.95(m, 2H), −0.03 (s, 9H) ppm. ESI-MS: m/z = 590.2 [M + H]^+^.

To a solution of (1s,4s)-1,4-dihydroxy-N,N-dimethylcyclohexane-1-carboxamide (507 mg, 2.71 mmol, 5 equiv) in DMF (4.00 ml) was added DBU (206 mg, 1.36 mmol, 2.5 equiv) and intermediate 13 (320 mg, 0.542 mmol, 1 equiv). The reaction was stirred at room temperature for 12 h. The solvent was partitioned between water and EtOAc. The aqueous layer was extracted with EtOAc (30.0 ml × 3) and the combined organic phases washed with brine, dried over MgSO_4_ and the solvent removed *in vacuo* to give the crude material. The product was purified by silica gel chromatography (0–10% CH_3_OH/CH_2_Cl_2_) to afford intermediate 14 as a white solid (105 mg, 0.150 mmol) in 28% yield. ESI-MS: m/z = 697.3 [M + H]^+^.

TBAF (1.0 M in THF) (2.00 ml, 2.00 mmol, 13 equiv) was added to a solution of intermediate 14 (105 mg, 0.150 mmol, 1 equiv) in THF (5.00 ml). The reaction was heated at 80°C for 3 h. The volatiles were removed *in vacuo* and the resultant residue was dissolved in MeOH (10.0 ml) and 2.5 M aq. sol. of NaOH (4.00 ml, 10.0 mmol, 67 equiv). The solution was stirred at room temperature for 4 h. Subsequently the volatiles were removed *in vacuo*, and the residue was taken up in water (10.0 ml) and adjusted to pH 7 with 2 M aq. sol. of HCl, then extracted with CH_2_Cl_2_. The combined organic extracts were washed with brine, dried over MgSO_4_ and the solvent removed *in vacuo*. The resultant crude material was purified by silica gel chromatography (0–20% CH_3_OH/CH_2_Cl_2_) to afford MSG012 (15.0 mg, 27.8 µmol) as an off-white solid in 19% yield (>99% pure as judged by HPLC). NMR data are consistent with the literature [40].

### Biological materials

For bacterial CaMMK2.1 expression, the human DNA sequence for truncated CaMKK2.1 (156–588), encompassing the kinase domain, was generated with a NH_2_-terminal GST-tag (PreScission cleavable) and COOH-terminal 6xHis-tag and cloned into pGEX-6P-1 using NdeI/BamHI restriction sites by Gene Universal (Newark, Delaware, United States). Other plasmids used in this study have been described previously [14,41]. pMT2-HA-γ1-R299G mutant construct was generated from pMT2-HA-γ1 [41] by site-directed mutagenesis using QuikChange site-directed mutagenesis kits (Stratagene) and sequence verified. MK-8722 (AOB33226) and PF-739 (AOB33584) were from AOBIOUS. AMP (A1752), ADP (A2754), ATP (A2383) and phenformin (P7045) were from Sigma–Aldrich.

### Mammalian cell culture

HEK293T/17, HEK293T, COS7, HeLa and PC3 cells (ATCC) were maintained in Dulbecco's Modified Eagle's medium (DMEM; Sigma–Aldrich) supplemented with 10% fetal bovine serum (FBS; Assay Matrix) at 37°C and 5% CO_2_. Primary hepatocytes from wild type mice were prepared using the collagenase perfusion method and cultured as described previously [42]. Cultures were incubated with fresh DMEM (no FBS) for 2 h prior to 1 h treatment with MSG011 (dissolved in DMSO) or phenformin (dissolved in H_2_O). Adherent HEK293T/17 cells at ∼50% confluency were triply transfected with AMPK α1 (pcDNA3.1 vector), β1 (COOH-terminal MYC fusion, pcDNA3.1 vector) and WT γ1 or γ1-R299G mutant (NH_2_-terminal HA fusion, pMT2 vector) in the presence of FuGENE HD transfection reagent (Promega) as per the manufacturers protocol [33]. Forty-eight hours post-transfection, cells were incubated ±2.5 µM MK-8722 for 60 min. Cells were harvested by first gently washing in ice cold phosphate buffered saline (PBS; Sigma–Aldrich), then scraping in ice cold lysis buffer (50 mM TRIS pH 7.5, 150 mM NaCl, 10% (v/v) glycerol, 50 mM NaF, 5 mM sodium pyrophosphate, 1% (v/v) Triton X-100, cOmplete protease inhibitor cocktail). Lysates were clarified by centrifugation (16 000***g***, 3 min, 4°C), flash frozen in liquid N_2_ and stored at −80°C until analysis.

### Metabolite extractions

HEK293T cell cultures grown in six-well plates were gently washed in ice cold PBS, lysed with 150 µl of ice cold 0.5 M perchloric acid (Univar) and clarified by centrifugation (16 000***g***, 3 min, 4°C). An amount of 75 µl of clarified lysate was neutralised with 25 µl of ice cold 2.3 M KHCO_3_ (Sigma–Aldrich), incubated on ice for 5 min and then centrifuged (16 000***g***, 3 min, 4°C) [16]. Supernatants were collected for analysis by liquid chromatography mass spectrometry–mass spectrometry (LC–MS/MS).

### Small molecule mass spectrometry

Adenine nucleotide metabolites were measured by LC–MS/MS with modifications to our previously described method [16]. A QTRAP 5500 mass spectrometer (AB Sciex) was operated with the turbo V ion source linked to Prominence UFLC_XR_ LC-20ADXR pumps (Shimadzu), SIL-20AC HT autosampler (Shimadzu) and CTO-20A HPLC column oven (Shimadzu). Both LC and MS instruments were controlled and managed with the Analyst 1.7.1 software (AB Sciex). Nitrogen was provided by a Genius NM3G nitrogen gas generator (PEAK Scientific). The autosampler was set at 4°C and column oven set at 30°C, which housed a 150 mm (length) × 0.5 mm (inner diameter) Hypercarb 3 µm porous graphitic carbon column (Thermo Fisher Scientific). The LC solvent system comprised of 50 mM triethylammonium bicarbonate buffer (TEAB, Sigma–Aldrich) pH 8.5 in pump A, and acetonitrile with 0.5% trifluoroacetic acid (TFA; Sigma–Aldrich) in pump B. A flow rate of 400 µl min^−1^ was used throughout a gradient program consisting of 0% B (2 min), 0 to 100% B (10 min), 100% B (3 min), 0% B (2 min). Data was analysed with MultiQuant 3.0.2 software (AB Sciex) using area under the LC curve. Calibration curves were determined by linear regression of the peak area ratio of each nucleotide and were required to have a correlation coefficient (*R*^2^) of >0.98. The MS and multiple reaction monitoring (MRM) values were optimised by separate infusion of 1 µg ml^−1^ solution in 50 mM TEAB at a flow rate of 50 µg ml^−1^ (Supplementary Table S2). All data were acquired in negative ion mode with the spray voltage set to −4500 V, source temperature set to 250°C, ion source gas 1 and 2 set at 30 and 60, respectively, curtain gas set at 20 and collision gas set to high. AEC was calculated from the ratios of [AMP], [ADP] and [ATP] (equation 1):

1 $$\hbox{AEC} = \displaystyle{{\lsqb {\rm{ATP}} \rsqb + \; \displaystyle{1 \over 2}\lsqb {\rm{ADP}} \rsqb } \over {\lsqb {\rm{ATP}} \rsqb + \lsqb {\rm{ADP}} \rsqb + \lsqb {\rm{AMP}} \rsqb }}\eqno\lpar 1\rpar $$

### Protein expression and purification of mammalian cell-expressed AMPK for kinase assays

Adherent HEK293T/17 cells at ∼50% confluency were triply transfected with full-length AMPK α1 or α2 (NH_2_-terminal GST fusion, pDEST27 vector), β1 or β2 (COOH-terminal FLAG fusion, pcDNA3.1 vector), and γ1, γ2 or γ3 (NH_2_-terminal HA fusion, pMT2 vector) in the presence of FuGENE HD transfection reagent (Promega) as per the manufacturers protocol [33]. Cells were harvested 48 hours post-transfection by first gently washing in ice cold phosphate buffered saline (PBS; Sigma–Aldrich), then scraping in ice cold lysis buffer (50 mM TRIS pH 7.5, 150 mM NaCl, 10% (v/v) glycerol, 50 mM NaF, 5 mM sodium pyrophosphate, 1% (v/v) Triton X-100, cOmplete protease inhibitor cocktail). Lysates were clarified by centrifugation (16 000***g***, 3 min, 4°C), flash frozen in liquid N_2_ and stored at −80°C until analysis. For radioactive kinase assays, AMPK was immobilised on glutathione Sepharose 4B resin (GE Healthcare) by incubation with lysates for 1.5 h at 4°C. Following centrifugation (1000***g***, 3 min, 4°C), the resin was washed twice in purification buffer (50 mM HEPES pH 7.4, 150 mM NaCl, 10% (v/v) glycerol, 0.02% (v/v) Tween-20), once in 50 mM HEPES pH 7.4 with 0.02% (v/v) Tween-20, then resuspended to a 50% slurry with 50 mM HEPES pH 7.4 and 10 µl added to the activity assays. For in solution kinase assays, α1β1γ1 AMPK was eluted from glutathione-Sepharose by incubating with purification buffer supplemented with 40 mM reduced glutathione (Sigma–Aldrich).

### Protein expression and purification of *E. coli*-expressed AMPK for kinase assays and crystallography

Recombinant full-length AMPK _6xHis_α2β1γ1 was expressed in *E. coli* Rosetta 2 (DE3) (Merck Millipore) after double-transformation of pET-Duet-1 (α and γ subunits) and pCOLA (β subunit) vectors [37]. Expression cultures were grown at 37°C in Luria-Bertani broth supplemented with 100 µg ml^−1^ ampicillin and 50 µg ml^−1^ kanamycin. Cultures shaking at 120 rpm in PYREX 2800 ml Fernbach-style culture flasks with baffles (Corning) were grown to an optical density (OD_600_) of 3.0 before induction with 500 µM isopropyl-β-d-1-thiogalactopyranoside (IPTG; Gold Biotechnology) and incubation overnight at 16°C. Cell pellets were resuspended in lysis buffer (50 mM TRIS pH 7.6, 500 mM NaCl, 5% (v/v) glycerol, 50 mM imidazole, 2 mM β-mercaptoethanol, 0.01 mM leupeptin, 0.1 mM AEBSF, 0.5 mM benzamidine hydrochloride), lysed using a precooled EmulsiFlex-C5 homogeniser (Avestin) and clarified via centrifugation (16 000***g***, 30 min, 4°C). Supernatant was passed through a HisTrap HP 5 ml Ni^2+^ column (GE Healthcare) at 1 ml min^−1^. The column was washed with 10 column volumes of chilled Ni^2+^ column buffer (50 mM TRIS pH 7.6, 500 mM NaCl, 10% (v/v) glycerol, 50 mM imidazole, 2 mM β-mercaptoethanol) before elution with Ni^2+^ column buffer supplemented with 400 mM imidazole. Proteinaceous fractions were then separated on a HiLoad 16/600 Superdex 200 gel filtration column (GE Healthcare) pre-equilibrated with size exclusion column buffer (SEC buffer; 50 mM TRIS pH 8.0, 150 mM NaCl, 2 mM tris(2-carboxyethyl) phosphine (TCEP)). AMPK containing fractions were pooled and concentrated to ∼2 mg ml^−1^ using Amicon centrifugal filter units (Merck Millipore) prior to CaMKK2 treatment.

Recombinant truncated GST-CaMKK2.1_156-588_-6xHis was expressed in *E. coli* Rosetta 2 (DE3) after transformation of the pGEX-6P-1 plasmid. Expression cultures were grown at 37°C in Luria-Bertani broth supplemented with 100 µg ml^−1^ ampicillin. Cultures shaking at 120 rpm in PYREX 2800 ml Fernbach-style culture flasks with baffles were grown to an OD_600_ of 0.8 before induction with 500 µM IPTG and incubation overnight at 16°C. Cell pellets were resuspended in lysis buffer (50 mM TRIS pH 7.6, 150 mM NaCl, 10% (v/v) glycerol, 2 mM BME, 1.0 mM NADP, 0.01 mM leupeptin, 0.1 mM AEBSF, 0.5 mM benzamidine hydrochloride), lysed using a precooled EmulsiFlex-C5 homogeniser (Avestin) and clarified via centrifugation (16 000***g***, 30 min, 4°C). Supernatant was passed through glutathione sepharose 4B (GSH4B) resin (GE Healthcare) at ∼1 ml min^−1^. The resin was washed with five column volumes (CV) of chilled GSH4B resin buffer (50 mM TRIS pH 7.6, 150 mM NaCl, 10% (v/v) glycerol, 2 mM BME), 5 CV of high salt GSH4B resin buffer (50 mM TRIS pH 7.6, 1 M NaCl, 10% (v/v) glycerol, 2 mM BME), and 10 CV of GSH4B resin buffer. The resin was resuspended in 3 CV of GSH4B resin buffer and GST tag removed by addition of 1 : 100 mass ratio 6xHis-PreScission protease (expressed and purified in-house from *E. coli*) and overnight incubation rotating at 4°C. The resin was pelleted by centrifugation (5000***g***, 5 min, 4°C) and supernatant containing CaMKK2.1_156-588_-6xHis collected and separated on a HiLoad 16/600 Superdex 200 gel filtration column pre-equilibrated with CaMKK2 SEC buffer (50 mM TRIS pH 7.6, 150 mM NaCl, 5% (v/v) glycerol, 2 mM TCEP). CaMKK2.1_156-588_-6xHis containing fractions were pooled and concentrated to ∼10 mg ml^−1^, flash frozen in L-N_2_ and stored at −80°C. The purified CaMKK2.1_156-588_-6xHis was analysed by time of flight-mass spectrometry (ToF-MS) and sample purity confirmed by SDS–PAGE.

To phosphorylate AMPK, purified protein was incubated in the presence of 0.5 mM ATP, 0.5 mM AMP, 2.5 mM MgCl_2_ (Merck Millipore), SEC buffer and purified CaMKK2 (1 : 2500 mass ratio of CaMKK2:AMPK; for 1 h at 22°C with gentle rolling. The phosphorylation reaction was terminated by directly loading onto a HiLoad 16/600 Superdex 200 gel filtration column pre-equilibrated with SEC buffer. Protein was either assayed or concentrated to ∼10 mg ml^−1^ using Amicon centrifugal filter units and flash frozen in L-N_2_ and stored at −80°C. The purified AMPK was analysed by ToF-MS and sample purity confirmed by SDS–PAGE.

### Radioactive kinase assays

AMPK activity was determined by phosphorylation of the SAMS peptide (Purar Chemicals, sequence: NH_2_-HMRSAMSGLHLVKRR-COOH) in a 25 µl reaction volume containing 100 µM SAMS peptide, 5 mM MgCl_2_, 200 µM ATP, [γ-^32^P]-ATP (PerkinElmer) and assay buffer (50 mM HEPES pH 7.4, 1 mM DTT and 0.02% (v/v) Tween-20) and purified AMPK. Phosphotransferase activity was conducted at 30°C for 10 min and reactions were quenched by spotting 15 µl onto phosphocellulose ion-exchange chromatography paper (made in-house), followed by repeated washes in 1% H_3_PO_4_ (Merck Millipore). Ultima Gold liquid scintillation fluid (5 ml; PerkinElmer) was added to vials containing dried phosphocellulose papers, and the level of ^32^P-transfer to the SAMS peptide was determined using a Tri-Carb 2900TR liquid scintillation counter (PerkinElmer).

### Immunoblotting

All samples were separated on 12% SDS–PAGE gels and transferred to an Immobilon-FL PVDF membrane (Merck Millipore). After blocking the membrane with 2% non-fat dry milk dissolved in PBS + 0.1% (v/v) Tween-20 (PBST; Sigma–Aldrich) for 30 min, membranes were incubated with primary antibodies (as indicated in Supplementary Table S3). Following repeated washes with PBST, fluorescently labelled secondary antibodies were added (as indicated in Supplementary Table S3), which were also dissolved in PBST. Immunoblots were visualised on the Odyssey Infrared Imaging System (LI-COR Biosciences) and immunoreactive bands analysed and quantified using Image Studio Software (LI-COR Biosciences).

### C2C12 oxygen consumption rate

Murine C2C12 myoblasts were plated at 1 × 10^4^ cells/well into Agilent Seahorse XFe24 Cell Culture Microplates in DMEM (life Technologies, Australia) containing 10% (v/v) fetal calf serum, 1% l-glutamine (v/v), and 1% (v/v) antibiotic solution (100 unit/ml penicillin/streptomycin) at 37°C in an atmosphere of 5% CO_2_. The next day, cells were switched to Seahorse basal assay media (Agilent, cat#: 102353-100; 48.5 ml) containing Glutamax (500 µl of 100×; Gibco, cat#35050), D-glucose (Sigma–Aldrich cat#: G8769) and sodium pyruvate (100×; Gibco cat#: 11360-070) at pH 7.4. MK-8722 at varying doses (0, 1, 10 µM) was incubated with myotubes 1 h prior to experiment, and the assay performed by titrating oligomycin (2 µM), FCCP (2 µM), and rotenone (2 µM) plus antimycin A (2 µM) as indicated. A Seahorse XFe24 Flux Analyzer (Seahorse Biosciences) was used to measure Oxygen Consumption Rate (OCR) at three time points during basal respiration and after each injection. Cells were lysed in equal volume (20 µl) and OCR measurements were normalised to total protein determined by polyacrylamide electrophoresis (5 µl lysate/well) and stain-free imaging.

### Crystallisation

Purified and phosphorylated _6xHis_α2β1γ1 was diluted to 4.0 mg ml^−1^ in SEC buffer and incubated on ice for 30 min with AMPK ligands; 5-fold molar excess of Ser108 peptide (NH_2_-KLPLTRSHNNFVARRR-COOH) [43], 3-fold molar excess of AMP, 1 : 1 molar ratio of staurosporine (Sigma–Aldrich) and 1 : 1 molar ratio of MSG011 [22]. The reservoir solution was dispensed into a 24-well VDX hanging drop plate (Hampton Research) and incubated at 22°C, the reservoir solution contained 8–10% (v/v) PEG 3350 (Sigma–Aldrich), 1% (v/v) glucose (Sigma–Aldrich), 0.1 M MgCl_2_, 0.1 M imidazole (Sigma–Aldrich, pH 6.2), 0.0005–0.003% (v/v) CAPB (AK Scientific). The protein/ligand mixture was added with the reservoir solution at a ratio of 1 : 1 at 22°C and crystals were grown for 1–2 weeks at 4°C via the hanging drop diffusion method. Protein crystals were then incubated with reservoir solution containing an additional 1–5% sucrose, 1–5% sorbitol, 1–5% glycerol, 1–5% ethylene glycol, 5% MPD and 1–5% PEG 400, for 1–2 min before flash-freezing in liquid nitrogen. Diffraction data were collected at the Australian Synchrotron (ANSTO) [44], processed using XDS [45] and scaled using AIMLESS in the CCP4 suite [46]. The PDB entry 6B1U [21] was used as a search model to solve the structure by molecular replacement using Phaser from the CCP4 suite [47]. Coot [48] and Buster [49] were used to perform iterative rounds of model building and refinement, respectively. The restraints and molecular coordinates for the new compounds were created using the PRODRG webserver. Omit maps where also calculated using Buster. Molprobity [50] was used to undertake structure validation and Pymol was used to create figures.

### Statistical analysis

All statistical analyses were performed using Prism 8 (GraphPad Software). Results from replicate experiments (n) were expressed as means ± standard error (SEM).

## Figures and Tables

**Scheme 1.**
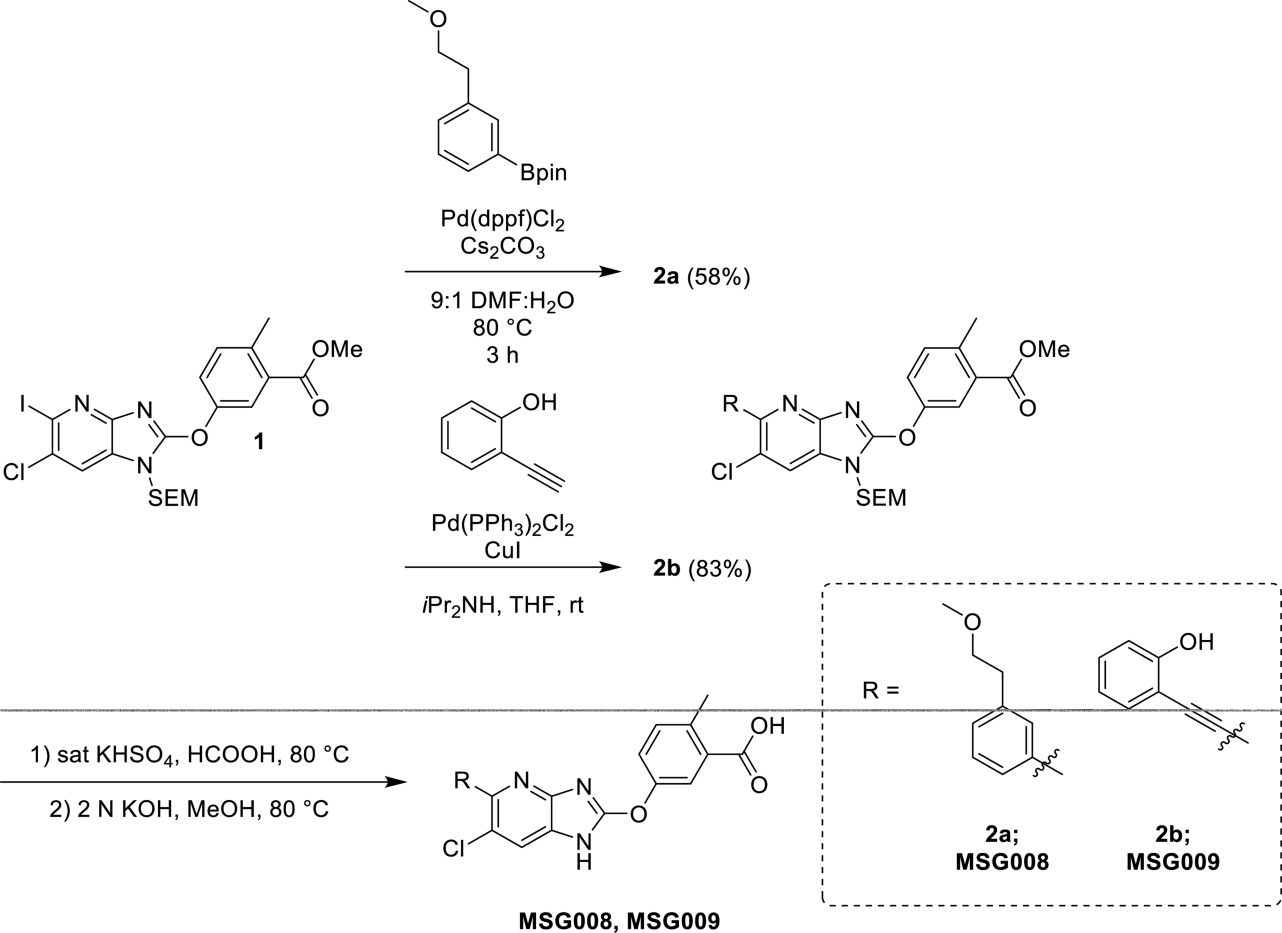
Synthesis route for MSG008 and MSG009.

**Scheme 2.**
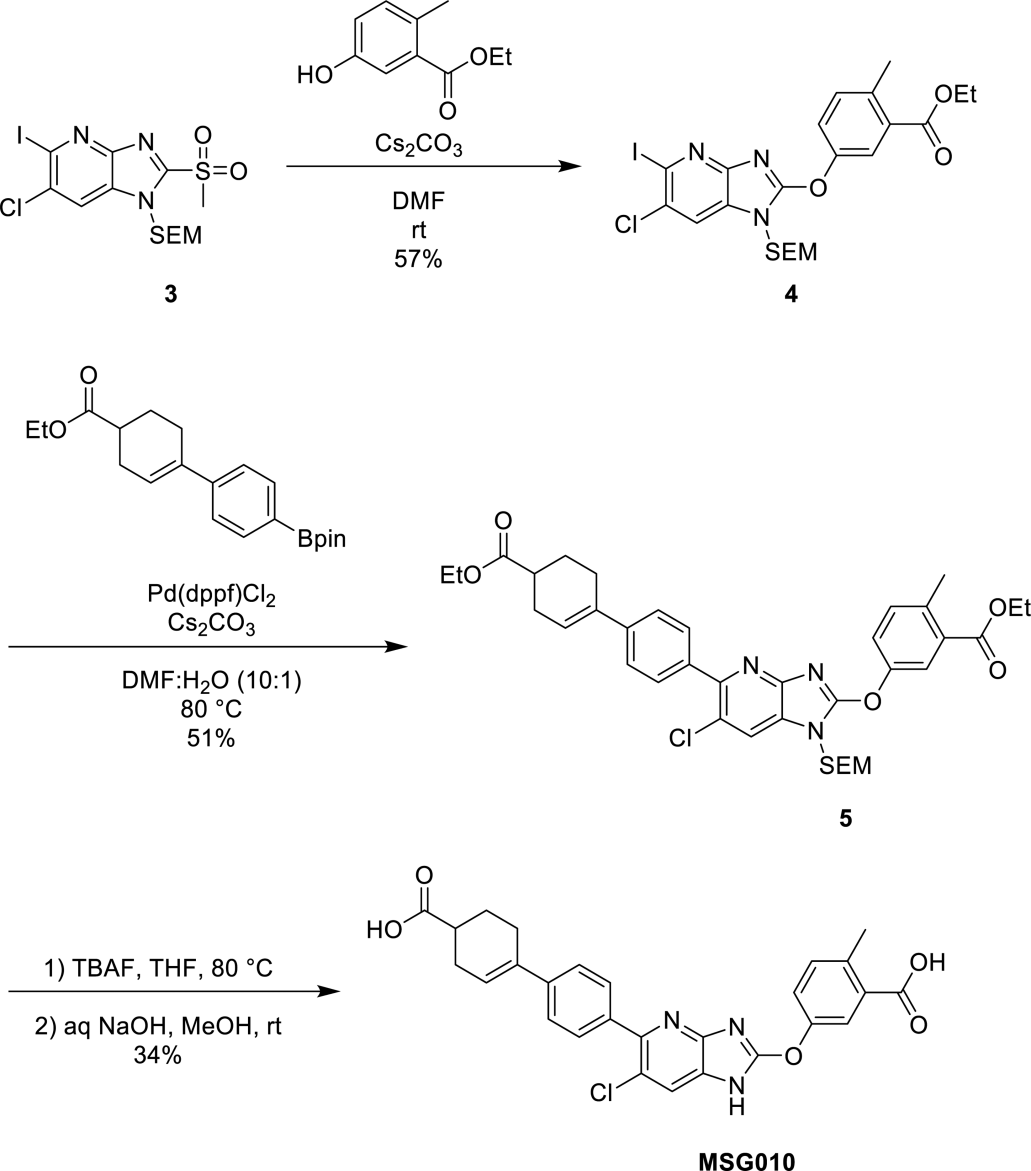
Synthesis route for MSG010.

**Scheme 3.**
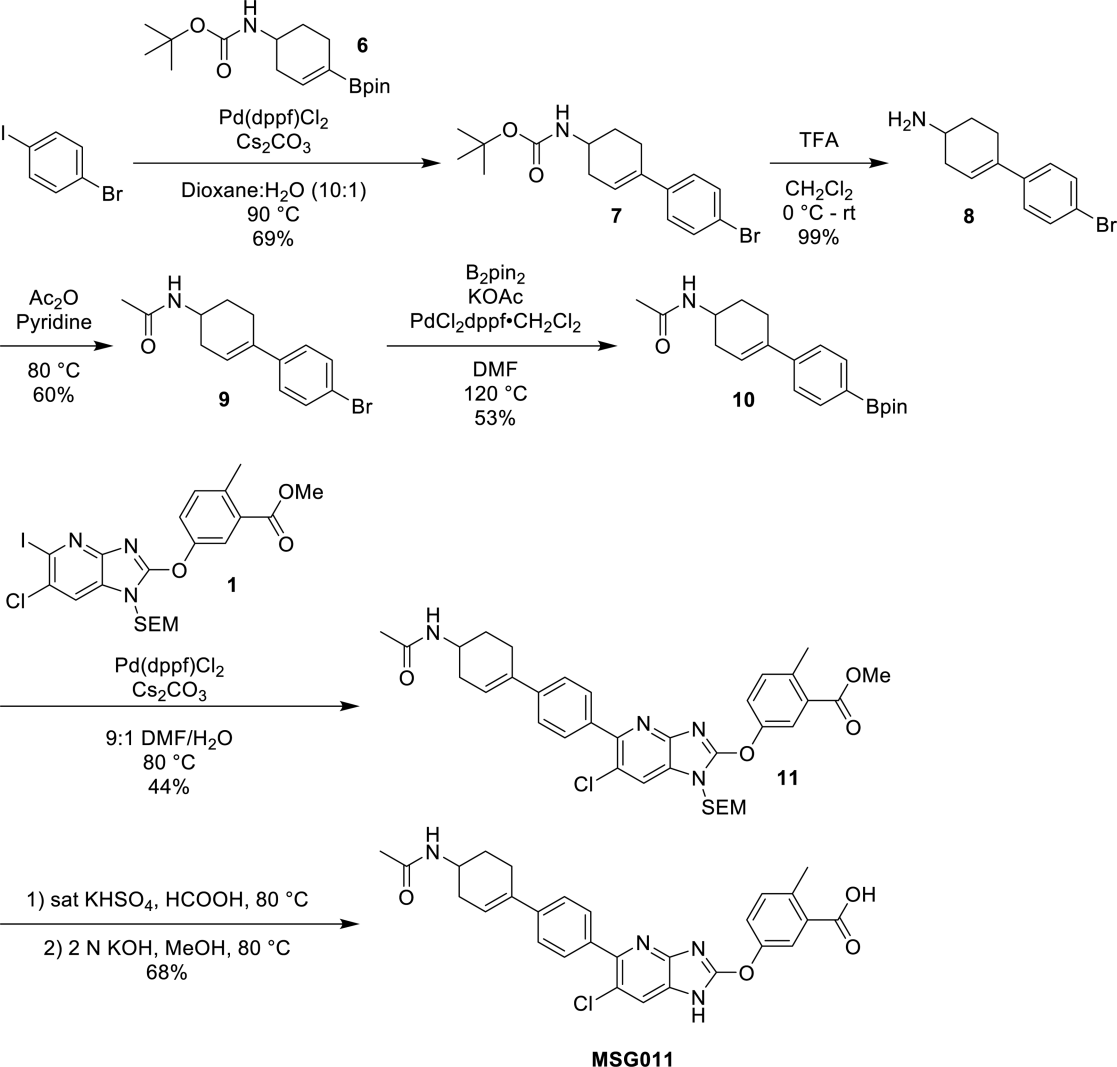
Synthesis route for MSG011.

**Scheme 4.**
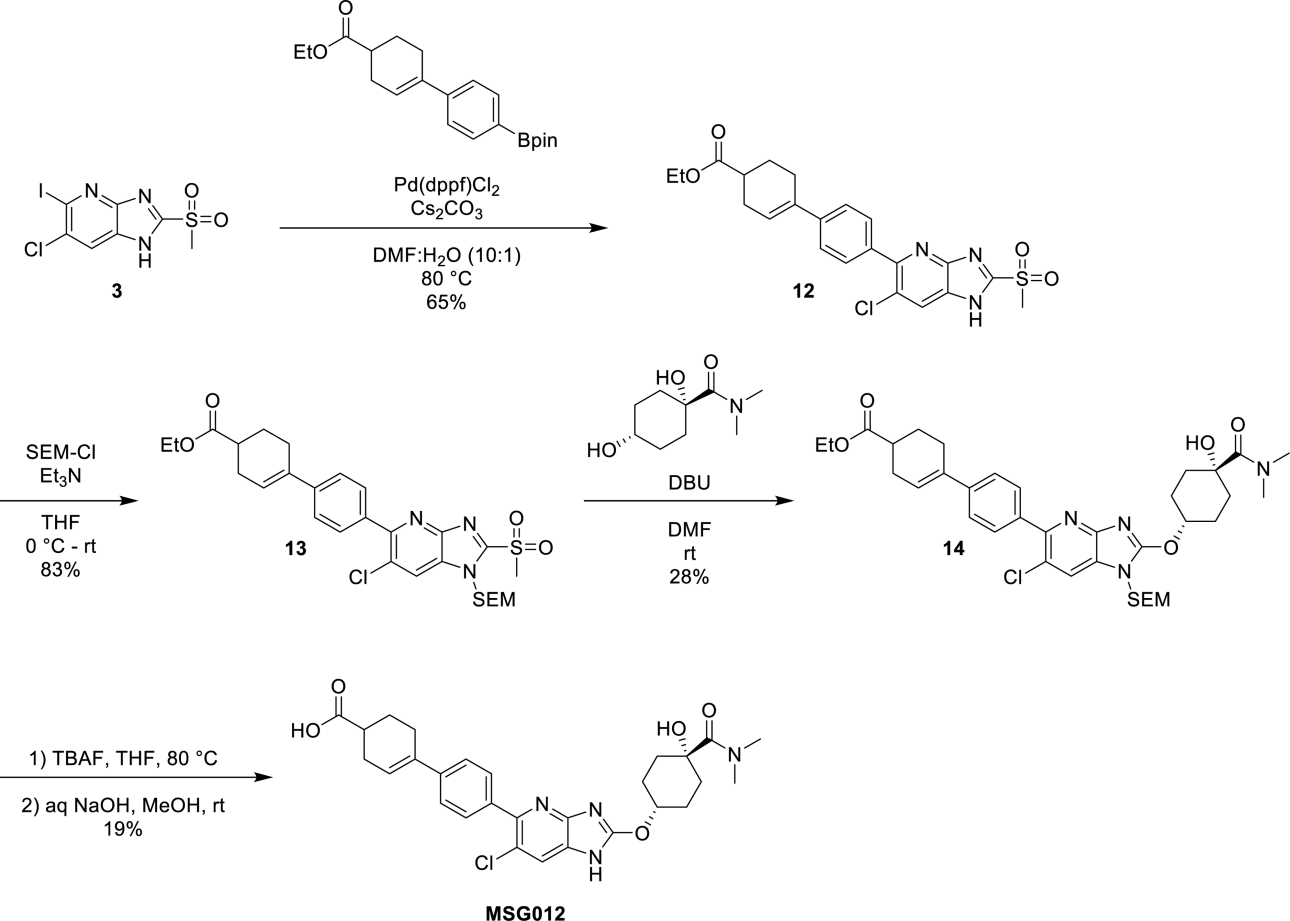
Synthesis route for MSG012.

## Data Availability

The co-ordinates for MSG011 complexed to α2β1γ1 have been deposited in the PDB under ID code 7MYJ.

## Abbreviations

ACC: acetyl-CoA carboxylase
ADaM: Allosteric Drug and Metabolite
AEC: adenylate energy charge
AMPK: AMP-activated protein kinase
CaMKK2: calcium/calmodulin-dependent kinase kinase 2
MRM: multiple reaction monitoring
OCR: oxygen consumption rate
T2DM: type 2 diabetes mellitus
