# Supplementary material for: Structure-function analysis of the AMPK activator SC4 and identification of a potent pan AMPK activator
Source: Biochem J. 2022 Jun 8;479(11):1181–204. doi: 10.1042/BCJ20220067 (PMC9317966; doi:10.1042/BCJ20220067)
Supplement: Supplementary Material [file BCJ-479-1181-s1.pdf]

Supplementary Material

Structure-function analysis of the AMPK activator SC4 and identification of a potent pan AMPK activator

Ashley J. Ovens[1,2,a], Yi Sing Gee[3,a], Naomi X.Y. Ling[1], Dingyi Yu[4], Justin P. Hardee[5], Jin D. Chung[5], Kevin R.W. Ngoei[4], Nicholas J. Waters[4], Nolan J. Hoffman[2], John W. Scott[4,6], Kim Loh[4], Katrin Spengler[7], Regine Heller[7], Michael W. Parker[8,9], Gordon S. Lynch[5], Fei Huang[10], Sandra Galic[4], Bruce E. Kemp[2,4], Jonathan B. Baell[3,10], Jonathan S. Oakhill[1,2] and Christopher G. Langendorf[4]

[1]Metabolic Signalling Laboratory, St. Vincent's Institute of Medical Research, Fitzroy 3065, Australia.
[2]Exercise and Nutrition Research Program, Mary MacKillop Institute for Health Research, Australian Catholic University, Melbourne 3000, Australia.
[3]Medicinal Chemistry, Monash Institute of Pharmaceutical Sciences, Monash University, Parkville 3052, Australia.
[4]Protein Chemistry and Metabolism, St. Vincent's Institute of Medical Research, Fitzroy 3065, Australia. [5]Centre for Muscle Research, Department of Anatomy and Physiology, The University of Melbourne, Melbourne, Victoria, 3010, Australia.
[6]The Florey Institute of Neuroscience and Mental Health, Royal Parade, Parkville 3052, Australia.
[7]Institute of Molecular Cell Biology, Center for Molecular Biomedicine, Jena University Hospital, 07745 Jena, Germany.
[8]ACRF Rational Drug Discovery Centre, St. Vincent's Institute of Medical Research, Fitzroy 3065, Australia.
[9]Structural Biology and Computational Design Laboratory, Department of Biochemistry and Pharmacology, Bio21 Molecular Science and Biotechnology Institute, University of Melbourne, Parkville, Victoria, Australia
[10]School of Pharmaceutical Sciences, Nanjing Tech University, No. 30 South Puzhu Road, Nanjing 211816, People's Republic of China.

[a]These authors contributed equally

Correspondence: Christopher G. Langendorf (clangendorf@svi.edu.au)

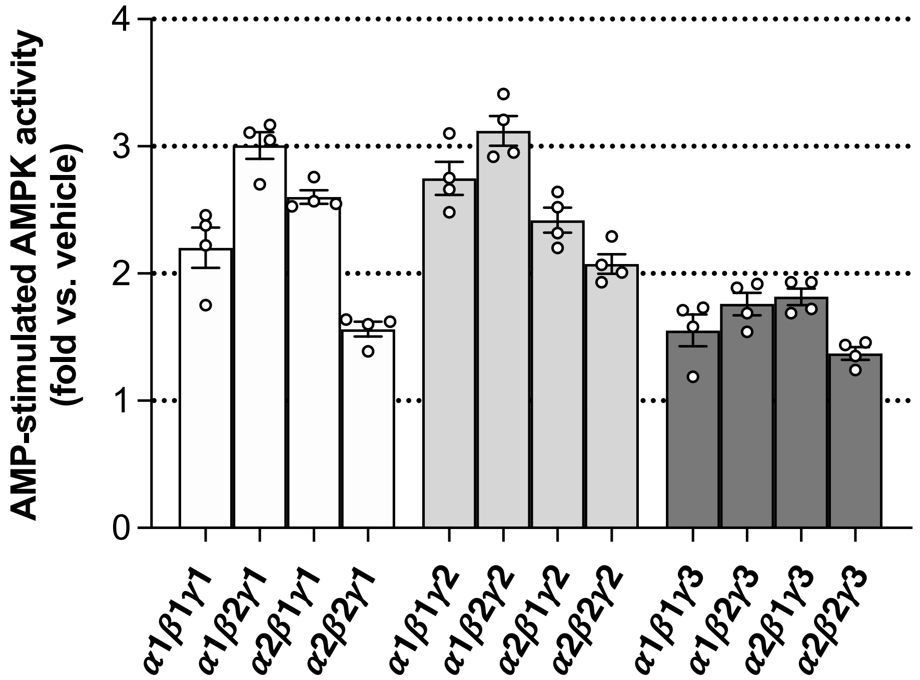

GST-AMPK complex	Specific activity (nmol.min^{-1}.mg^{-1} enzyme)	Fold activation (+ 100 μM AMP)
α1β1γ1	105.6	2.2
α1β2γ1	67.5	3.0
α2β1γ1	7.2	2.6
α2β2γ1	9.1	1.6
α1β1γ2	195.8	2.7
α1β2γ2	280.9	3.1
α2β1γ2	9.7	2.3
α2β2γ2	19.7	2.1
α1β1γ3	58.0	1.6
α1β2γ3	81.0	1.8
α2β1γ3	9.1	1.8
α2β2γ3	13.1	1.4

Supplementary Figure 1. Validation of mammalian cell AMPK preparations. Related to Figure 1. Allosteric activation of AMPK complexes by AMP confirms integrity of the protein preparations used in this study. GST-tagged AMPK was expressed in HEK293T/17 cells, immobilised on glutathione Sepharose and activities measured ±100 μM AMP. n = 4, data presented as mean fold change in AMPK activity relative to vehicle ± SEM. Basal activities for each complex are displayed in the associated table.

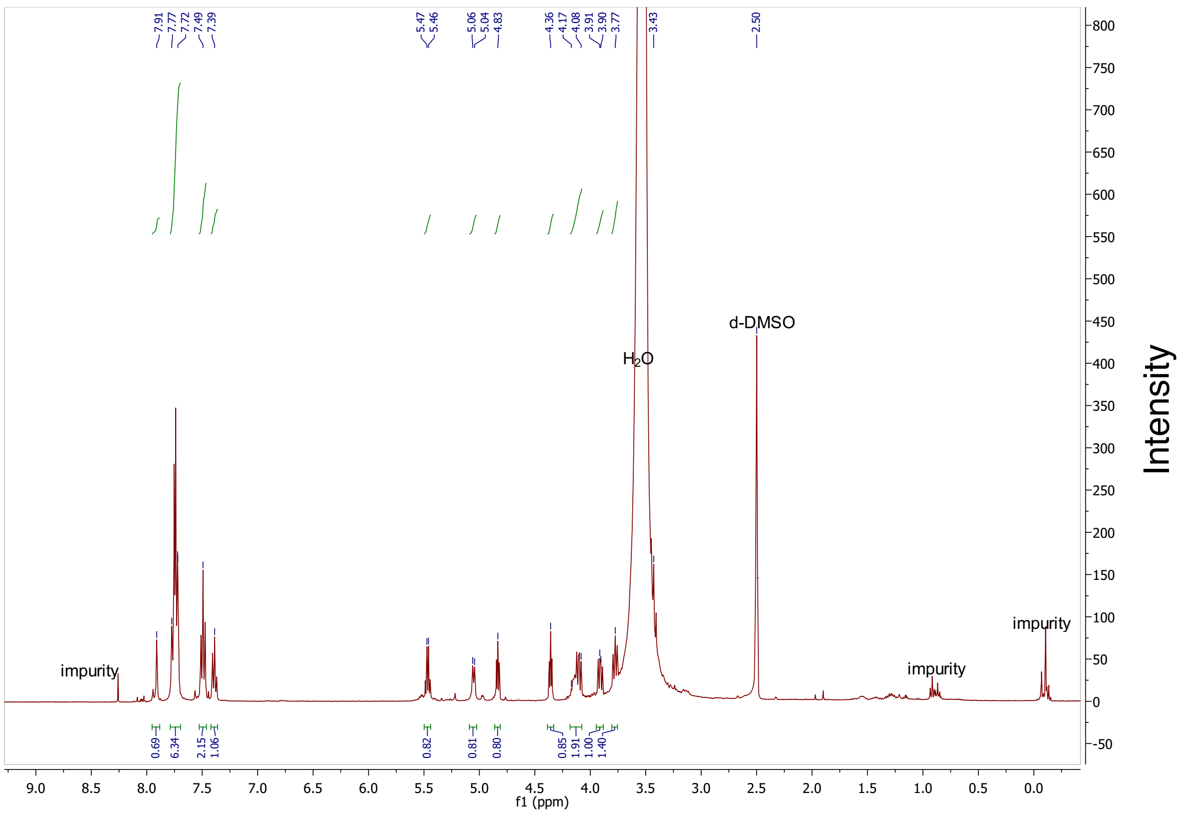

Supplementary Figure 2. NMR validation of MK-8722. Related to Figure 2. NMR data are consistent with the literature.

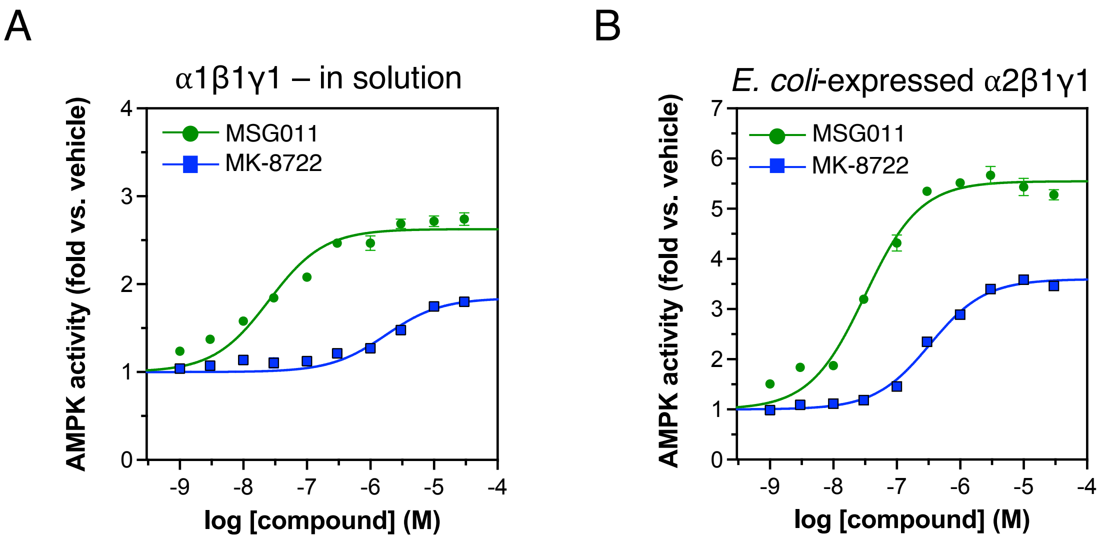

Compound	*α1β1γ1* – in solution	
	EC_{50} (nM)	Max. fold activation
MSG011	25.2	2.6
MK-8722	1744	1.8

Compound	*E. coli*-expressed *α2β1γ1*	
	EC_{50} (nM)	Max. fold activation
MSG011	32.6	5.5
MK-8722	333.6	3.6

Supplementary Figure 3. In solution activation kinetics for AMPK complexes. Related to Figure 2. Activities measured in the presence of 0-30 µM AMPK activators. Dose-response curves for MSG011 and MK-8722 activation of (A) HEK293T/17-expressed GST-α1β1γ1, eluted from glutathione Sepharose, and (B) *E. coli*-expressed myristoylated his-α2β1γ1. n = 3-6, data presented as mean fold AMPK activation relative to vehicle ± SEM.

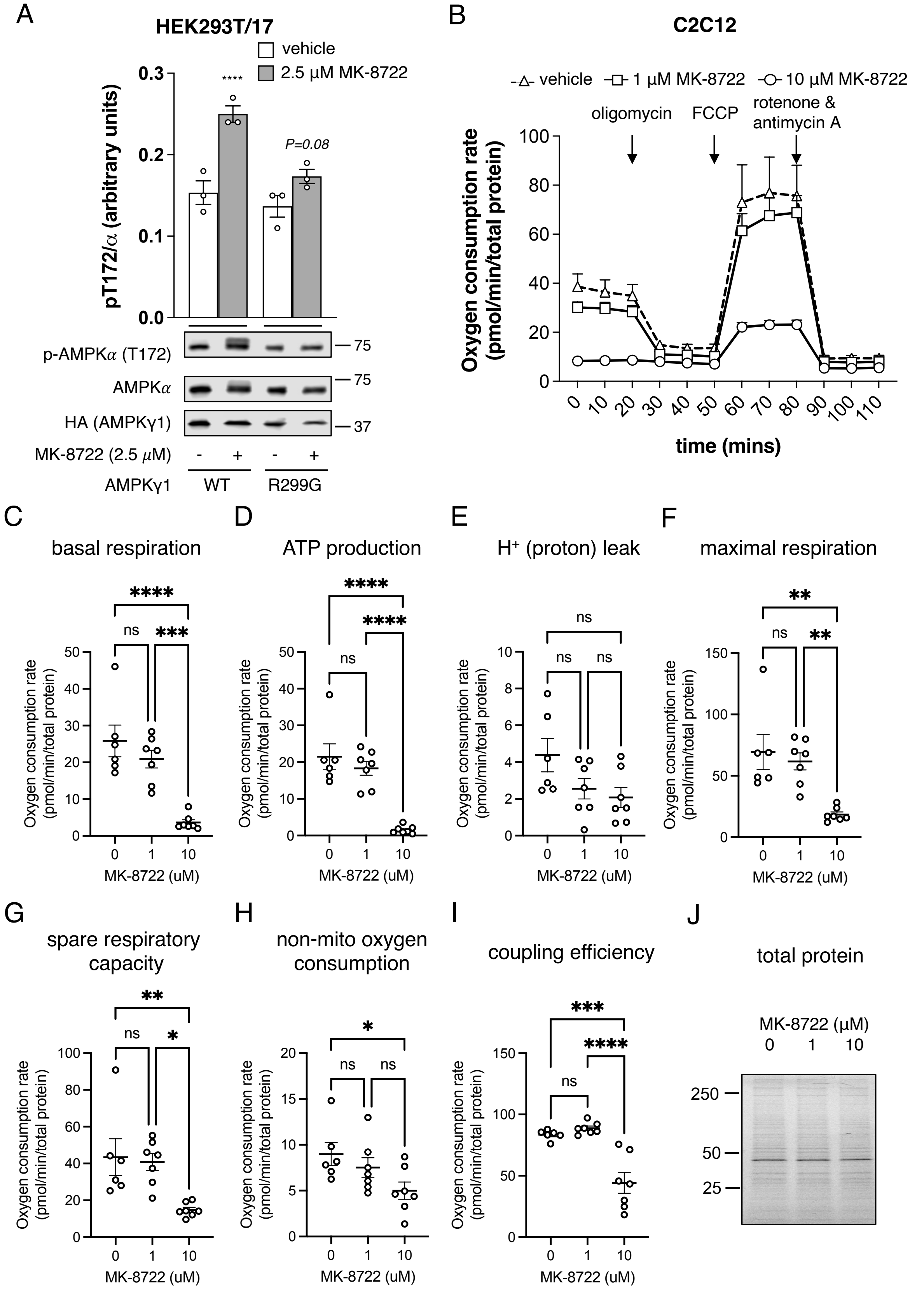

Supplementary Figure 4. Dose-dependent suppression of mitochondrial function following MK-8722 treatment. (A) HEK293T/17 cells, transiently expressing WT γ1AMPK or the AMP-insensitive γ1 mutant R299G, were incubated with 2.5 µM MK-8722 for 60 min.

Lysates were assessed for phosphorylation of AMPKα-Thr172 by immunoblot. n = 3, data presented as mean T172 phosphorylation (arbitrary units) ± SEM. Statistical significance vs. respective vehicle treatment was performed using unpaired, two-tailed Student's t-test. **** $P < 0.0001$ indicates significant increase in α-Thr172 phosphorylation relative to vehicle treated WT γ1AMPK. Representative immunoblots are shown. (B) C2C12 myoblast oxygen consumption rate following treatment of MK-8722 (0, 1, 10 μM) for 1 h and titration of specific respiration modulators. (C-I) Parameters of mitochondrial function. n = 6-7, data presented as mean oxygen consumption rate ± SEM. Statistical analysis was performed by one-way ANOVA with post hoc Tukey's multiple comparison test. * $P < 0.05$, ** $P < 0.01$, *** $P < 0.001$ and **** $P < 0.0001$ indicate significant difference to 0 μM MK-8722. (J) Lysates were assessed for total protein content following the respiration assay.

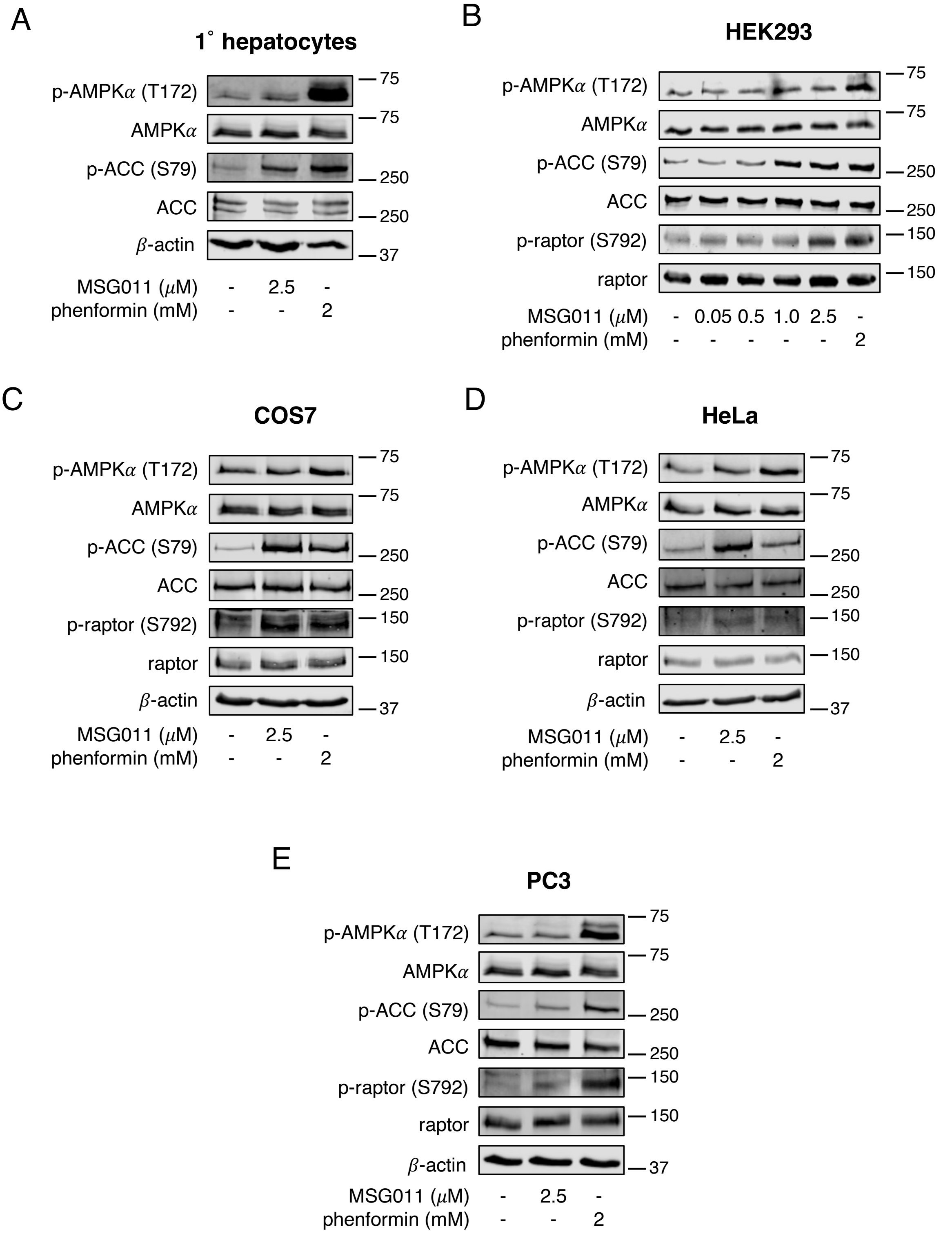

Supplementary Figure 5. Representative immunoblots for cellular markers of AMPK signalling following MSG011 treatment. Related to Figure 3. Lysates prepared from (A) mouse primary hepatocytes, (B) HEK293T, (C) COS7, (D) HeLa and (E) PC3 cells, treated with 0-2.5 μM MSG011 (1 h) or 2 mM phenformin (1 h), were assessed for phosphorylation of AMPKα-Thr172, ACC-Ser79 or raptor-Ser792 by immunoblot. Immunoblots shown are representative of 4 independent experiments.

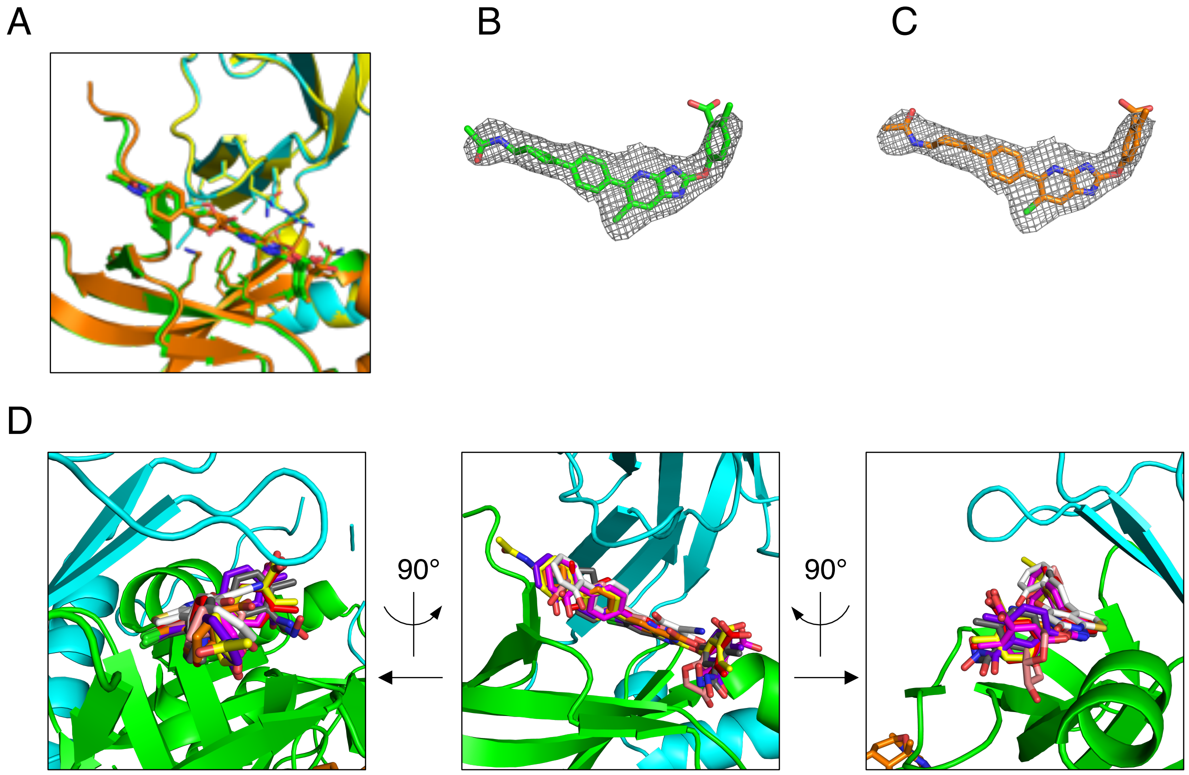

Supplementary Figure 6. MSG011 binds at the ADaM site of AMPK. Related to Figure 4. (A) Close up cartoon representation of MSG011 bound to the ADaM site of α2β1γ1 heterotrimer 1 (α2 and MSG011, green; β1, cyan) superposed (CBM aligned) with heterotrimer 2 (α2 and MSG011, orange; β1, yellow) in the asymmetric unit. Critical hydrophobic and polar residues that interact with MSG011 shown as sticks. Omit density (F_o-F_c) maps for MSG011 in heterotrimer 1 (B) and heterotrimer 2 (C) are shown contoured to 2.5 sigma and coloured grey. (D) MSG011 in the α2β1γ1 structure (α2, green; β1, cyan; MSG011, yellow) was superposed (CBM aligned) with previously solved AMPK structures of ADaM site compounds SC4 (PBD: 6B1U; α2β1γ1, magenta), A769662 (PBD: 4CFF; α2β1γ1, light grey), 991 (PBD: 4CFE; α2β1γ1, red), PF739 (PBD: 5UFU; α1β1γ1, salmon), and PF-06409577 (PBD: 5KQ5; α1β1γ1, orange), R734 (PBD: 6C9F; α1β1γ1, dark grey) and R739 (PBD: 6C9G; α1β1γ1, purple). Left and right images are 90° rotations of the middle image.

Supplementary Table 1. Data collection and refinement statistics.

**Data Collection**	
**Resolution range (Å)**	47.79 - 2.95 (3.055 - 2.95)
**Space group**	P 1 21 1
**Unit cell**	75.84 134.2 141.82 90 92.55 90
**Total reflections**	144589 (10250)
**Unique reflections**	59194 (5846)
**Multiplicity**	2.4 (2.2)
**Completeness (%)**	98.91 (97.82)
**Mean I/sigma(I)**	10.7 (2.2)
**Wilson B-factor**	71.05
**R-merge**	0.053 (0.370)
**R-meas**	0.075 (0.523)
**R-pim**	0.053 (0.369)
**CC1/2**	0.995 (0.735)
**Refinement**	
**Reflections used in refinement**	59163 (5845)
**Reflections used for R-free**	2990 (287)
**R-work**	0.2344 (0.3476)
**R-free**	0.2587 (0.3796)
**Number of non-hydrogen atoms**	14774
**macromolecules**	14630
**ligands**	144
**Protein residues**	1880
**RMS (bonds)**	0.012
**RMS (angles)**	1.63
**Ramachandran favoured (%)**	99.07
**Ramachandran allowed (%)**	0.93
**Ramachandran outliers (%)**	0.00
**Rotamer outliers (%)**	0.00
**Clashscore**	4.98
**Average B-factor**	76.08
**macromolecules**	76.26
**ligands**	57.50

Statistics for the highest-resolution shell are shown in parentheses.

Supplementary Table 2. MRM Values for AMP, ADP and ATP.

Metabolite	Mass (Da) Q1	Quantifier Mass (Da) Q3	Qualifier Mass (Da) Q3	Declustering Potential (DP)	Entrance Potential (EP)	Collision Energy (CE)	Collision Cell Exit Potential (CXP)	Dwell Time (msec)
**AMP**	346.06	79	-	-100	-10	-62	-11	300
	-	-	97	-100	-10	-24	-11	50
**ADP**	426.03	79	-	-100	-10	-62	-13	200
	-	-	134	-100	-10	-62	-17	50
**ATP**	505.99	159	-	-100	-10	-52	-43	100
	-	-	79	-100	-10	-50	-25	50

Supplementary Table 3. Antibodies used for immunoblotting.

Antibody (Catalogue Number)	Type	Source	Incubation Time	Dilution	Supplier
**AMPKα (2793)**	Primary	Mouse	Overnight	1:1000	Cell Signaling Technology
**AMPKα pThr172 (2535)**	Primary	Rabbit	Overnight	1:1000	Cell Signaling Technology
**ACC pSer79 (3661)**	Primary	Rabbit	Overnight	1:1000	Cell Signaling Technology
**Raptor (2280)**	Primary	Rabbit	Overnight	1:1000	Cell Signaling Technology
**Raptor pSer792 (2083)**	Primary	Rabbit	Overnight	1:1000	Cell Signaling Technology
**IRDye 680RD-labeled Streptavidin (926-68079)**	Primary	Mouse	1 hour	1:12,500	LI-COR Biosciences
**IRDye 680RD (926-68070)**	Secondary	Goat (anti-mouse IgG)	1 hour	1:10,000	LI-COR Biosciences
**IRDye 680RD (926-68071)**	Secondary	Goat (anti-rabbit IgG)	1 hour	1:10,000	LI-COR Biosciences
**IRDye 800CW (926-32210)**	Secondary	Goat (anti-mouse IgG)	1 hour	1:10,000	LI-COR Biosciences
**IRDye 800CW (926-32211)**	Secondary	Goat (anti-rabbit IgG)	1 hour	1:10,000	LI-COR Biosciences
